# Morphology-Dependent SnO_2_ Supported Ru Catalysts for Catalytic Oxidation of Vinyl Chloride Emission

**DOI:** 10.3390/nano16140850

**Published:** 2026-07-10

**Authors:** Hongyu Cui, Mingju Wang, Maosheng Zhou, Tianqi Cao, Junyi Liu, Chuanhui Zhang

**Affiliations:** 1 Institute of Materials for Energy and Environment, College of Materials Science and Engineering, Qingdao University, Qingdao 266071, China; 2 Faculty of Chemical Engineering and Energy Technology, Shanghai Institute of Technology, Shanghai 201418, China

**Keywords:** volatile organic compounds, catalytic oxidation, support morphology, surface active oxygen species, reaction pathway and mechanism

## Abstract

Constructing catalytic materials with specific morphologies is an effective approach to boosting and optimizing their catalytic performances. Herein, SnO_2_ supports with diverse morphologies of nanosphere, nanosheet and nanorod were separately synthesized for the fabrication of supported Ru catalysts. Catalytic evaluation results for vinyl chloride (VC) oxidation reveal that the nanosphere-shaped catalyst (Ru/SnO_2_-Sp) exhibits the optimum catalytic activity (achieving 90% of VC conversion at 268 °C), exceptional long-term catalytic durability, and cyclic stability comparable to nanosheet-shaped and nanorod-shaped catalysts (Ru/SnO_2_-Sh and Ru/SnO_2_-Rd). Additionally, Ru/SnO_2_-Sp presents promising applicability with regard to its impressive resistance behavior towards carbon dioxide and water vapor interference. Characterization results clearly demonstrate the close structure–activity relationship, primarily depending on the physicochemical parameters of specific surface area, redox capacity, surface oxygen species and valence state distribution of Ru. In situ infrared spectroscopy clarifies the key catalytic pathways of VC oxidation over Ru/SnO_2_-Sp, evidencing that the enol species from C-Cl bond cleavage and initial activation of VC molecules and the carboxylic acid species resulting from the subsequent oxidation of enol are both recognized as the crucial organic intermediates.

## 1. Introduction

Volatile organic compounds (VOCs) refer to organic chemicals having melting points below room temperature and boiling points in the range of 50 to 260 °C. VOCs possess hazardous properties such as carcinogenicity, teratogenicity and mutagenicity, enabling them to be a key target for atmospheric pollution control during China’s 15th Five-Year Plan period [1,2]. Particularly, chlorinated volatile organic compounds (CVOCs), mainly coming from industrial production processes and the preparation of chemical raw materials, show higher toxicity, more resistance to degradation, and pose more challenging treatment requirements compared to conventional low-chain aliphatic VOCs. CVOCs have caused non-negligible harm to the atmospheric environment and human health [3,4]. For instance, trichloroethane might have a negative impact on the human central nervous system. Additionally, most CVOCs are also regarded as being closely related to human cancers. In particular, trichloroethylene and vinyl chloride (VC) were categorized as Group 1 carcinogens by the IARC of the World Health Organization in 2014 and 2012, respectively [5,6]. Consequently, finding effective techniques for CVOC removal is currently a significant focus in the environmental protection field.

Thermal catalytic oxidation can efficiently eliminate CVOCs and possesses advantages such as low operating temperature, wide applicability, high treatment efficiency and absence of secondary pollution [7]. Currently, the catalysts for catalytic oxidation of CVOCs can be mainly classified into three categories: supported noble metals [8], transition metal oxides [9,10] and zeolites [11]. Among them, supported noble metal catalysts have become a research focus due to their remarkable redox capability and excellent catalytic oxidation performance [12]. Common noble metals such as Pt, Pd, Ru, Rh and Au are frequently employed as the active components in supported catalysts [13,14,15,16]. Although these catalysts are capable of catalytically oxidizing CVOCs at relatively low temperatures (100–300 °C), their poor chlorine resistance not only results in severe catalyst deactivation but also produces abundant hypertoxic polychlorinated organics and even dioxins [17,18]. In comparison, noble metal Ru exhibits significant advantages. On one hand, it has a relatively low economic cost. Throughout the Deacon reaction, Ru active domains can catalytically convert adsorbed chlorine species into chlorine gas (HCl + O_2_ → Cl_2_ + H_2_O), effectively preventing the issue of chlorine poisoning [19,20]. This enables the Ru-based catalyst to demonstrate excellent catalytic stability in CVOCs’ catalytic oxidation [6,21]. On the other hand, Ru possesses exceptional redox capability because of its multiple valence states (i.e., Ru^0^, Ru^2+^ and Ru^4+^) and electronic coordination environments. During catalytic oxidation of CVOCs, these characteristics enable efficient activation of reactive oxygen species within the reaction system, enhancing its oxidative attacking capacity toward hydrocarbon fragments and promoting their complete degradation [22]. Accordingly, this facilitates a thorough and complete progression of the catalytic oxidation reaction. For instance, Liu et al. found that loading Ru onto Co_3_O_4_ can increase the quantity of reactive oxygen species on the catalyst, which further enhances the efficiency for the catalytic oxidation of 1,2-dichloroethane [23]. It has been shown that the high catalytic stability of Ru-based catalysts in CVOCs’ oxidation is closely associated with the dechlorination capacity of Ru species through the Deacon process. To be precise, the Deacon process is centered on the formation reaction of chlorine gas, including the adsorption and conversion of chlorine species on the surface of the catalyst, which is frequently employed to evaluate the chlorine poisoning resistance of catalysts. Gu et al. also pointed out in their studies on the catalytic oxidation of CVOCs over Ru/Ce*_x_*Al*_γ_* [24], Ru/CeO_2_ [25], and Ru/TiO_2_ [26] that these Ru-based catalysts exhibited sustainable catalytic activity. Thus, it can be seen that Ru, with favorable redox capability and resistance to chlorine poisoning, displays outstanding catalytic behaviors, making it a highly promising catalyst for CVOCs’ oxidation.

The support is able to affect the catalytic activity of a metal catalyst via the strong metal–support interaction effect (SMSI) [27,28,29]. The catalytic oxidation activity and durability of catalysts in multiple reactions can be significantly adjusted through targeted modification of the support, such as CO oxidation [30,31], CH_4_ oxidation [32,33] and VOC oxidation.

Tin dioxide (SnO_2_), as an n-type metal oxide, exhibits abundant surface defects and reactive lattice oxygen species [34,35], and it has garnered widespread attention due to its modifiable surface properties, high oxygen mobility and excellent thermal stability [36]. For instance, Liu et al. adopted SnO_2_ with a large specific surface area as the support, and a large number of exposed reactive oxygen sites and acid sites on the support promote the deep oxidation of toluene [37]. The morphological features of the support have a significant impact on the catalytic performance of the catalyst. For example, Hu et al. prepared supported Pd catalysts with different morphologies of SnO_2_ supports to investigate their catalytic activity for methane combustion [38]. The results showed that the Pd/SnO_2_-NRs catalyst had the highest content of reactive Pd^2+^ species and surface-adsorbed oxygen. The strongest interaction between Pd and SnO_2_-NRs supports finally realized efficient catalytic combustion of methane.

Nevertheless, there is a distinct lack of research focusing on morphology-regulated SnO_2_-supported Ru catalysts for the catalytic degradation of VC. Long et al. verified that RuO_2_ thin films can undergo epitaxial growth on SnO_2_ surfaces, generating strong interfacial electronic metal–support interaction via Ru-O-Sn linkages. Electron transfer occurs from Sn to Ru, which yields abundant metallic Ru^0^ active sites, greatly facilitating the cleavage of C–Cl bonds in CVOCs and enabling their complete catalytic oxidation. Furthermore, Ru species anchored on SnO_2_ serve as dedicated dechlorination sites, lowering the desorption temperature of chlorine species and markedly improving the long-term chlorine resistance of the catalyst [39]. Ding et al. found that RuO_2_ could be constructed on the SnO_2_ surface based on the lattice-matching principle due to their similar crystal structures, enabling highly dispersed RuO_2_ on the rutile-phase SnO_2_ support. RuO_2_ exhibits selective capture ability toward chlorine species, which safeguards the oxygen vacancies responsible for oxidative reactions and thereby promotes the catalytic oxidation of VOCs [12,40]. Nevertheless, all the above-mentioned studies only employed irregular bulk SnO_2_ without targeted morphological regulation of supports. To date, few investigations have systematically compared SnO_2_ supports with nanosphere, nanorod and nanosheet structures, making it impossible to distinguish the synergistic regulatory effects of morphology on the content of surface reactive oxygen species, the strength of metal–support interaction, and the conversion efficiency of VC. This critical research gap motivates the present work to systematically uncover the morphology-dependent structure–activity relationship of Ru/SnO_2_ catalysts during vinyl chloride oxidation.

In this work, SnO_2_ with variable morphologies, namely as nanosphere, nanorod and nanosheet, were separately synthesized as supporting materials for the preparation of supported Ru catalysts. Extensive experimental and characterization approaches were applied to study the morphological influence of SnO_2_ supports on VC oxidation and the physical and chemical properties of the synthesized Ru/SnO_2_ catalysts. Additionally, the reaction intermediates and their conversion pathways were analyzed by in situ infrared spectroscopy for unveiling a plausible surface reaction mechanism on Ru/SnO_2_ catalysts. This work offers fresh perspectives on the design and advancement of high-performance catalytic materials for the catalytic oxidation of CVOCs.

## 2. Experimental Section

### 2.1. Catalyst Preparation

SnO_2_ supports with variable morphologies namely as nanosheet, nanorod and nanosphere, were all synthesized by a hydrothermal method but using different conditions. For a simple description, these SnO_2_ supports were designated as SnO_2_-Sh, SnO_2_-Rd and SnO_2_-Sp, respectively, and the detailed preparation procedures are described as follows.

SnO_2_-Sh: A total of 1.899 g of SnCl_4_·5H_2_O (Aladdin Reagents Co., Ltd., Shanghai, China), 0.64 g of cetyltrimethyl ammonium bromide (CTAB, Adamas Reagent Co., Ltd., Shanghai, China) and 1.25 g of NaOH (Sinopharm Chemical Reagent Co., Ltd., Shanghai, China) were dissolved in 35 mL of deionized water and then stirred for 1 h. Next, the solution was sonicated for 30 min to guarantee uniformity. After that, the solution was transferred to a Teflon-lined autoclave and hydrothermally treated at 180 degrees Celsius for 10 h. The precipitate was collected by centrifugation, washed completely, dried overnight at 60 °C, and finally calcined at 500 °C for 3 h in air to get the SnO_2_-Sh support.

SnO_2_-Rd: A total of 1.06 g of SnCl_4_·5H_2_O was dissolved in 10 mL of deionized water, and then 30 mL of NaOH aqueous solution (1.08 mol L^−1^) was added drop by drop while continuously stirring for 0.5 h. Then, 40 mL of ethanol (Sinopharm Chemical Reagent Co., Ltd., Shanghai, China) was added, and the mixture was stirred for another 0.5 h. Next, the mixture was transferred to a Teflon-lined autoclave and hydrothermally reacted at 200 °C for 72 h. The precipitate was centrifuged and washed, dried overnight at 60 °C, and finally calcined at 500 °C for 3 h in air to get the SnO_2_-Rd support.

SnO_2_-Sp: A total of 0.76 g of SnCl_4_·5H_2_O was added to a mixed solution of deionized water and ethanol with a volume ratio of 1:10, and then 2 mL of concentrated hydrochloric acid (mass fraction 36.5–38%, Sinopharm Chemical Reagent Co., Ltd., Shanghai, China) was added. The solution was then sonicated for 30 min to ensure homogeneity. Then, the solution was transferred to a Teflon-lined autoclave and hydrothermally treated at 200 °C for 24 h. The precipitate was collected via centrifugation and washed repeatedly, dried overnight at 60 °C, and finally calcined at 500 °C for 3 h in air to get the SnO_2_-Sp support.

SnO_2_-supported Ru catalysts were prepared using a deposition–precipitation method. The preparation details are described as follows. A total of 0.5 mL of RuCl_3_ solution (0.02 g_Ru_ mL^−1^, Heraeus (China) Investment Co., Ltd., Shanghai, China) was diluted in 20 mL of deionized water. A total of 1.0 g of SnO_2_ support was added into the RuCl_3_ solution and evenly dispersed under continuous stirring. A diluted ammonia solution (0.5 mol L^−1^) was added drop by drop to the previous mixture while stirring until the pH value reached 8.0. After continuous stirring for 3 h, the precipitate was separated by centrifugation and washed thoroughly. After drying overnight at 60 °C, the collected solid was treated at 400 °C for 1.5 h in a 10% H_2_/Ar atmosphere. According to the relative ratio between RuCl_3_ and SnO_2_, the nominal loading percentage of Ru on the SnO_2_ support was 1.0 wt.%, and the obtained catalysts were simply designated as Ru/SnO_2_-Sh, Ru/SnO_2_-Rd and Ru/SnO_2_-Sp, respectively.

### 2.2. Catalyst Characterizations

A variety of characterization methods were employed to study the physical and chemical characteristics of SnO_2_ supports and Ru/SnO_2_ catalysts. In situ diffuse reflection infrared Fourier transform spectroscopy (in situ DRIFTs, Thermo Fisher Scientific, Waltham, MA, USA) was used to monitor the adsorption routes and conversion evolutions of VC on catalyst surfaces. The complete experimental parameters for these characterizations are given in the Supplementary Materials.

### 2.3. Catalytic Performance Evaluation

The catalytic oxidation of VC was examined in a fixed-bed reactor. A total of 0.2 g of catalyst screened to 40–60 mesh was placed into the quartz tube. The total flow rate of the mixed reaction gas was 50 mL min^−1^, which was composed of 0.1 vol.% VC and 8 vol.% oxygen with Ar as the balance gas. A Fuli 9790 II gas chromatograph equipped with a flame ionization detector (FID) was utilized to measure the inlet and outlet gas concentrations in real-time, and the VC conversion was calculated by using the following equation:

$$X_{\mathrm{VC}}\;=\;\frac{{\left[\mathrm{VC}\right]}_{\mathrm{in}}-{\left[\mathrm{VC}\right]}_{\mathrm{out}}}{{\left[\mathrm{VC}\right]}_{\mathrm{in}}}\times 100\%$$

Herein, ${\left[\mathrm{VC}\right]}_{\mathrm{in}}$ and ${\left[\mathrm{VC}\right]}_{\mathrm{out}}$ represent the VC concentration at the inlet and outlet, respectively.

## 3. Results and Discussion

### 3.1. XRD and Raman Analysis

**Figure 1:**
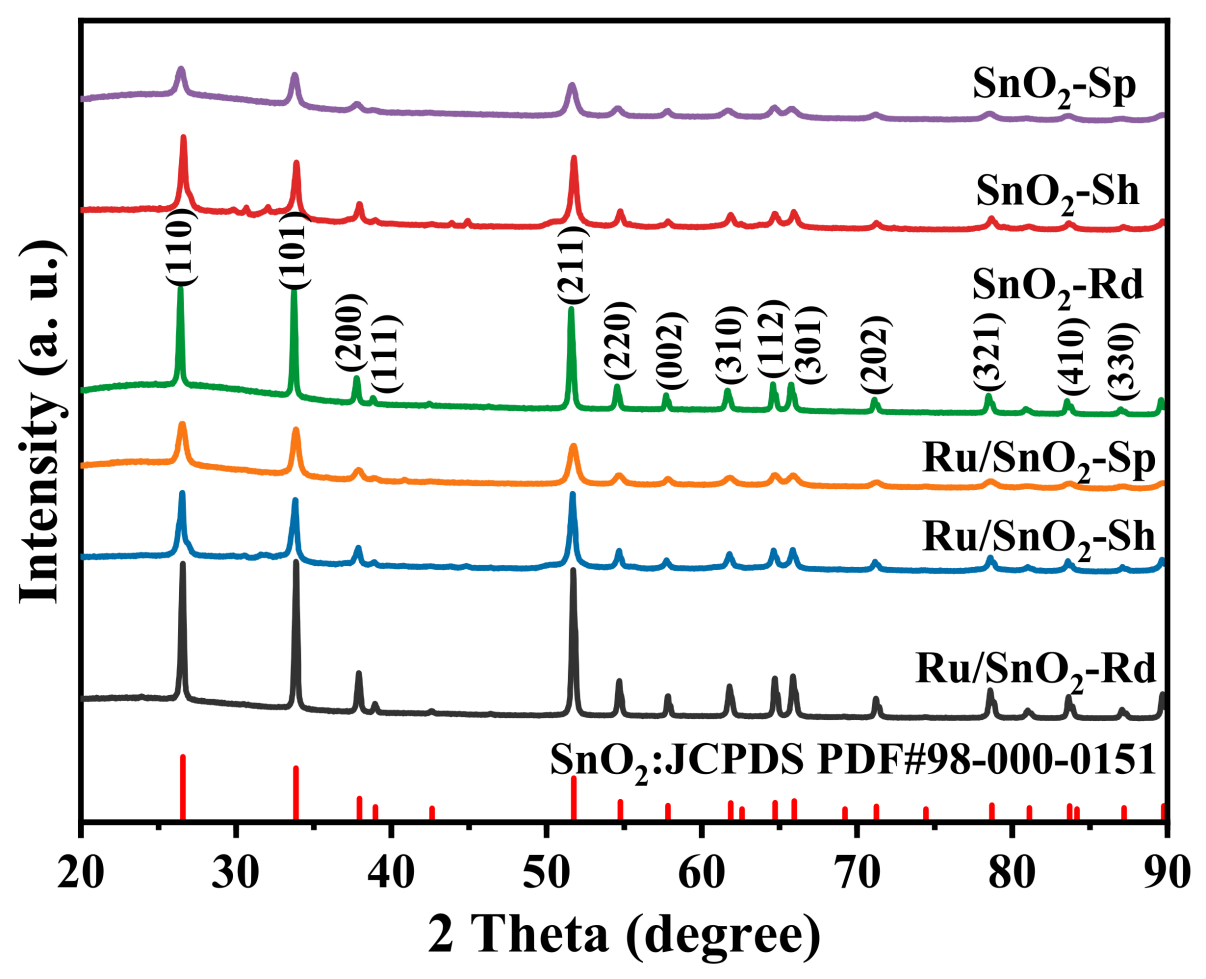
XRD patterns of SnO_2_ supports and Ru/SnO_2_ catalysts.

Figure 1 displays the XRD patterns of SnO_2_ supports and Ru/SnO_2_ catalysts. A series of diffraction peaks can be observed, located at 26.58°, 33.86°, 37.94°, 38.97°, 51.76°, 54.74°, 57.82°, 61.87°, 64.72°, 65.94°, 71.77°, 78.71°, 84.31° and 87.65° on each SnO_2_ support, which typically correspond to the (110), (101), (200), (111), (211), (220), (002), (310), (112), (301), (202), (321), (410) and (330) facets of tetragonal rutile-structured SnO_2_ (JCPDS PDF#98-000-0151), respectively. This indicates that all prepared SnO_2_ supports exhibit the polycrystalline characteristics [41]. In addition, it is noteworthy that the intensity of diffraction peaks for SnO_2_-Rd is notably higher than those over SnO_2_-Sp and SnO_2_-Sh, which reflects its higher crystallinity. It is speculated that the crystallinity difference can be correlated with the regulatory effect of preparation conditions (i.e., hydrothermal temperature) on the crystal growth of SnO_2_. After the loading of Ru onto the supports, the obtained Ru/SnO_2_ catalysts exhibit the similar diffraction peaks as the pristine SnO_2_ supports but show no diffraction signal assignable to metallic Ru or RuO_2_, which reveals the low Ru loading or good surface dispersion of Ru species on the supports [12,42]. Interestingly, one can note that each Ru/SnO_2_ catalyst shows a relatively higher intensity of diffraction peaks in comparison with its corresponding SnO_2_ support. This phenomenon may originate from the incorporation of Ru species into the SnO_2_ lattice. Given the similar ionic radii and chemical characteristics of Ru and Sn, it is speculated that Ru atoms may either substitute Sn lattice sites or occupy lattice interstices [43]. Consequently, this would reduce the lattice defects in SnO_2_, promote ordered crystal arrangement, and thus enhance the crystallinity of the catalysts.

**Figure 2:**
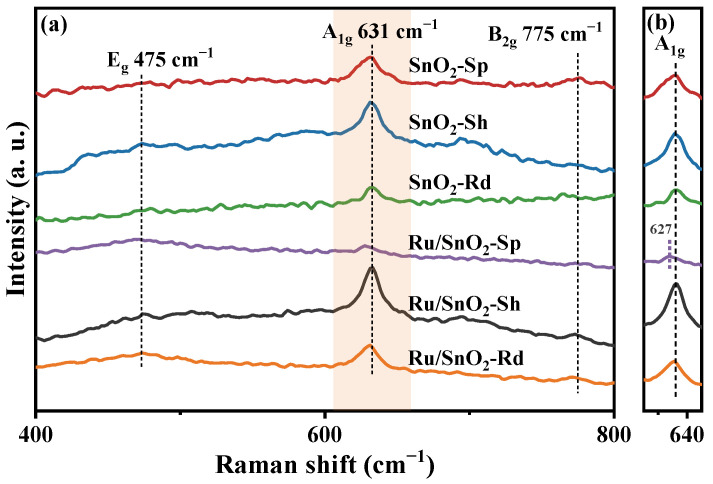
Raman spectra of SnO_2_ supports and Ru/SnO_2_ catalysts. (**a**) Full-range Raman spectra from 400 to 800 cm^−1^; (**b**) magnified view of the A_1g_ characteristic peak region. The orange shaded area represents the spectral region magnified in panel (**b**) and the dash lines denote the positions for Raman peak assignment.

Figure 2 displays the Raman spectra of SnO_2_ supports and Ru/SnO_2_ catalysts for the purpose of more investigation into their structural properties. As shown in Figure 2a, all SnO_2_ supports and Ru/SnO_2_ catalysts exhibit similar vibration peaks, which are well consistent with the typical Raman features of rutile SnO_2_. Specifically, three Raman bands are observed at 775, 631 and 475 cm^−1^, corresponding to the B_2g_, A_1g_ and E_g_ vibrational modes of rutile SnO_2_, respectively [44]. Amongst these, E_g_ represents a doubly degenerate vibrational mode, while A_1g_ and B_2g_ are non-degenerate modes, which correspond to the symmetric and asymmetric stretching oscillatory motions of the Sn–O bonds, respectively.

As precisely shown in Figure 2b, the specific peak corresponding to the A_1g_ vibrational mode is located around 631 cm^−1^. After Ru loading, the positions of the A_1g_ peaks for catalysts show a slight shift. Raman peak position is fundamentally governed by the Sn–O bond vibrational frequency, which is positively dependent on the bond force constant. The substitution of lattice Sn atoms by Ru atoms or the interstitial incorporation of Ru into the SnO_2_ lattice induces noticeable lattice distortion, which elongates the Sn–O bonds and reduces their force constants, accompanied by the generation of substantial oxygen vacancies. The decreased bond force constant effectively reduces the overall lattice vibration frequency, thereby leading to the red shift of the A_1g_ characteristic peak. This structural evolution is highly consistent with the aforementioned XRD results [45]. As shown in Figure 2b, the Ru/SnO_2_-Sp catalyst exhibits the largest peak shift with a deviation of up to 4 cm^−1^.

This can be attributed to the fact that Ru/SnO_2_-Sp possesses strengthened metal–support interaction, which drives substantial electron transfer from SnO_2_ to Ru and triggers severe Sn–O lattice distortion. The charge imbalance of the lattice further induces abundant oxygen vacancies. The combined effects markedly reduce the Sn–O bond force constant, thus yielding the largest red shift in Raman spectra. Additionally, the Raman spectra exhibit no characteristic peaks related to Ru or RuO_2_ species, which further substantiates the remarkable surface dispersion of Ru species on the SnO_2_ support.

### 3.2. Morphological Characteristics and Porosity

**Figure 3:**
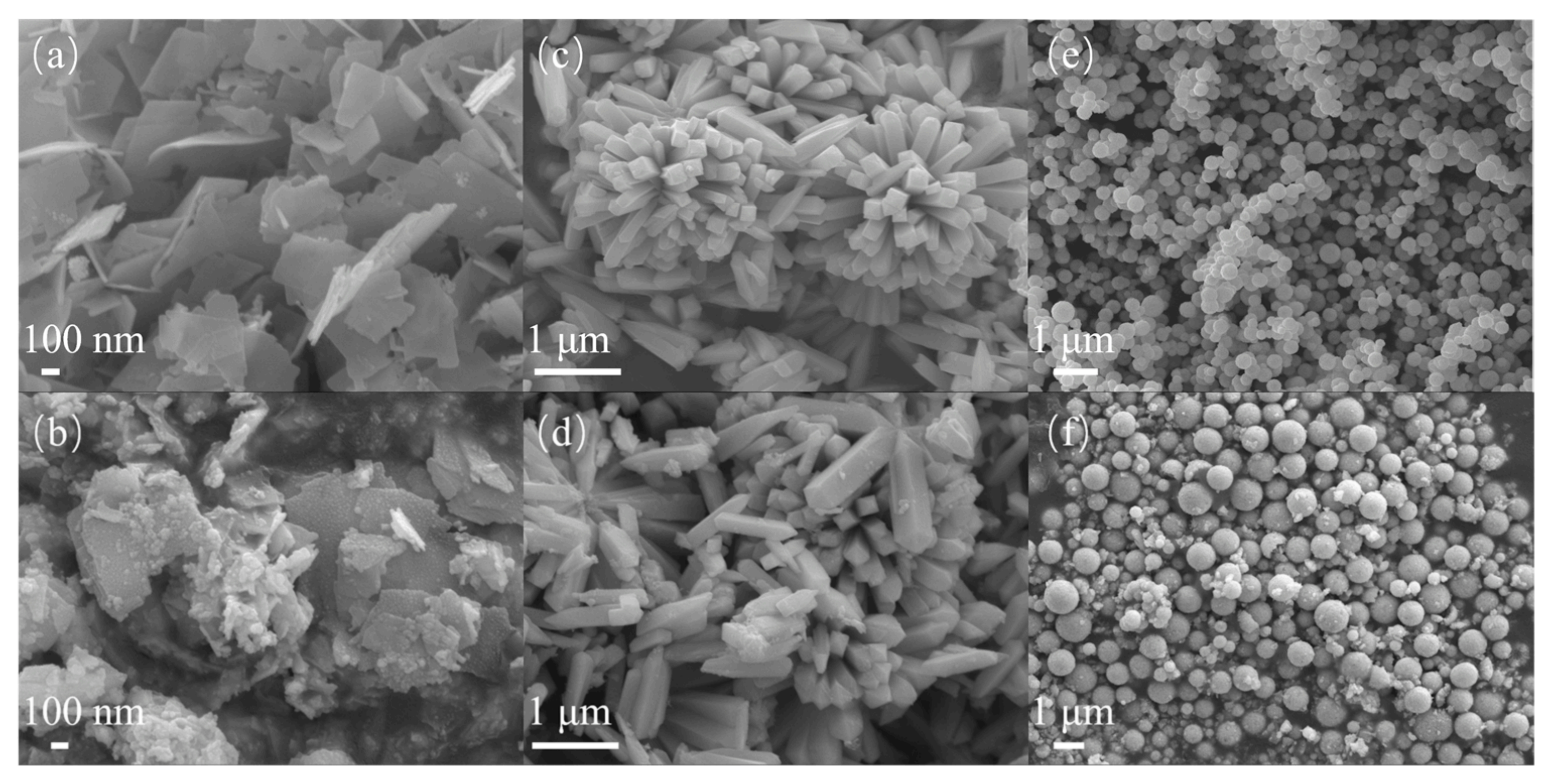
SEM images of (**a**) SnO_2_-Sh, (**b**) Ru/SnO_2_-Sh, (**c**) SnO_2_-Rd, (**d**) Ru/SnO_2_-Rd, (**e**) SnO_2_-Sp and (**f**) Ru/SnO_2_-Sp.

Figure 3 shows the SEM images of SnO_2_ supports and Ru/SnO_2_ catalysts. In Figure 3a, one can observe that SnO_2_-Sh exhibits a flat sheet-like structure with uneven lengths ranging from approximately 200 to 800 nm. Figure 3c shows the nearly square cross-section over SnO_2_-Rd, and the nanorods are densely arranged and aggregated into a flower-like structure. Figure 3e shows that SnO_2_-Sp possesses obviously uniform microsphere characteristics with a relatively smooth surface and spherical diameter of ca. 300 nm. On the basis of Figure 3b,d,f, it can be noticed that the Ru/SnO_2_ catalysts exhibit a morphology similar to that of their corresponding SnO_2_ supports. This implies that the surface loading of Ru has minimal impact on the original microstructure of the supports, allowing them to well retain their original morphological characteristics. It is inferred that the structural stability of SnO_2_ support is crucial for the maintenance of catalytic performance over the Ru/SnO_2_ catalyst since the micro-morphology alterations typically result in catalytic activity decay in catalytic reactions. Meanwhile, this finding also suggests that there may be a relatively mild interaction between Ru and the SnO_2_ support, enabling Ru to disperse evenly on the surface of SnO_2_ without disturbing its structure.

**Figure 4:**
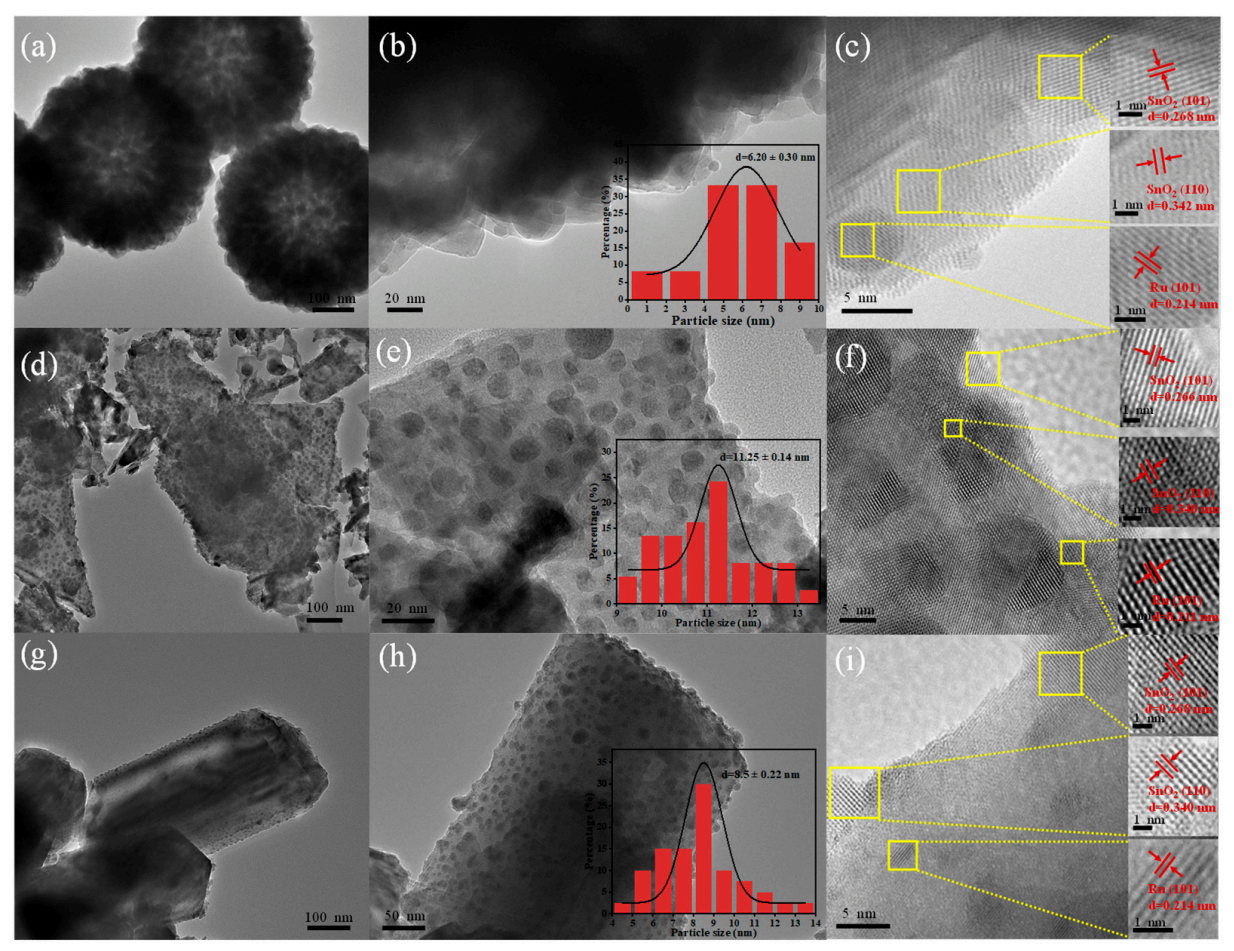
TEM and HR-TEM images of (**a**–**c**) Ru/SnO_2_-Sp, (**d**–**f**) Ru/SnO_2_-Sh and (**g**–**i**) Ru/SnO_2_-Rd.

Figure 4 shows the TEM and high-resolution TEM (HR-TEM) images of Ru/SnO_2_ catalysts, exhibiting identical structural features to those seen in SEM images. This further confirms that the loading of Ru does not alter the intrinsic morphological features of the SnO_2_ supports. In Figure 4c,f,i, it can be seen that the lattice fringes having an interplanar spacing of 0.268 nm is associated with the (101) crystal plane of rutile SnO_2_, while the fringe displaying a 0.340 nm lattice spacing matches the (110) crystal plane [42]. In Figure 4b,e,h, Ru nanoparticles with the average size in the range of 6–11 nm are observed to be highly dispersed on the surface of Ru/SnO_2_ catalysts, and the average particle size is determined to be 6.20 ± 0.30, 11.25 ± 0.14 and 8.50 ± 0.22 nm over Ru/SnO_2_-Sp, Ru/SnO_2_-Rd and Ru/SnO_2_-Sh, respectively. Notably, the Ru/SnO_2_-Sp catalyst exhibits the smallest average size of Ru particles with uniform surface dispersion, thus enabling it to expose a greater number of active sites [46]. Furthermore, the lattice fringes with a spacing of 0.214 nm are detectable in each Ru/SnO_2_ catalyst, which match the (101) crystal plane of metallic Ru (Ru^0^) [47]. This suggests that Ru^0^ species represent the primary active phase of the present catalysts.

**Figure 5:**
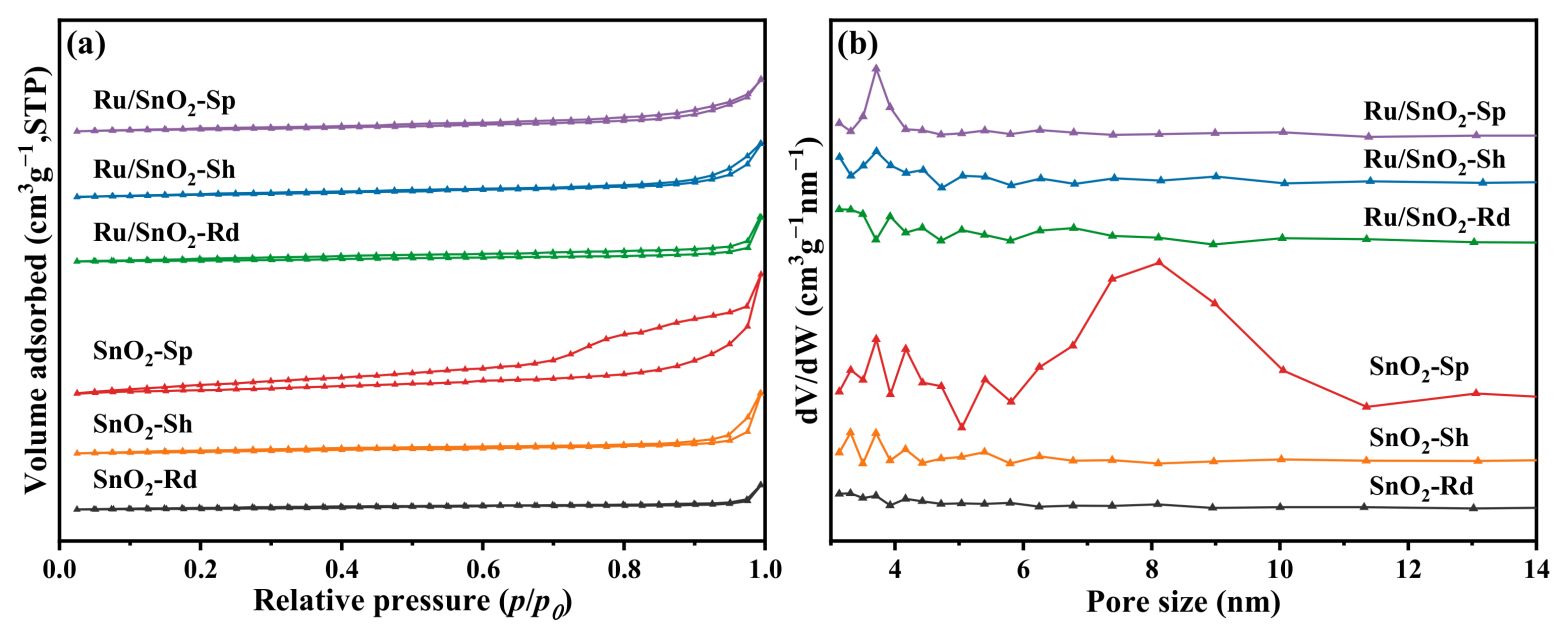
(**a**) The adsorption–desorption isotherms of N_2_ and (**b**) the pore size distributions of SnO_2_ supports and Ru/SnO_2_ catalysts.

As shown in Figure 5a, both of the SnO_2_-Sp and SnO_2_-Sh supports display type IV adsorption isotherms and H4 hysteresis loops in the relative pressure range of 0.6 < *p*/*p*_0_ < 1.0, which implies the presence of a mesoporous structure over both supports [48]. Combined with Figure 5b, it can be clearly seen that the pore size of SnO_2_-Sp and SnO_2_-Sh supports is primarily distributed within the range of 2–12 nm, further confirming their mesoporous feature. In contrast, SnO_2_-Rd shows an extremely weak hysteresis loop in the enlarged image in Figure S1, which suggests its poorly developed porous structure and is consistent with its low specific surface area in Table 1. Generally, the high specific surface area of a catalyst provides abundant active sites, facilitating the adsorption and desorption kinetics and thereby enhancing the catalytic performance. As shown in Table 1, it is noted that SnO_2_-Sp possesses a higher specific surface area, pore volume and diameter comparable to SnO_2_-Sh and SnO_2_-Rd, thereby providing ample space and unobstructed channels for gaseous molecule adsorption. The specific surface areas of SnO_2_-Sh and SnO_2_-Rd supports are 13.8 and 6.3 m^2^ g^−1^, respectively. After Ru loading, the values increase to 17.9 and 8.6 m^2^ g^−1^ for Ru/SnO_2_-Sh and Ru/SnO_2_-Rd, respectively. This phenomenon is mainly attributed to the surface structure reconstruction induced by Ru introduction, which further exposes more accessible active sites. On the contrary, the specific surface area of Ru/SnO_2_-Sp decreased from 30.4 to 20.2 m^2^ g^−1^ compared with the SnO_2_-Sp support, owing to the filling of some pores of the SnO_2_ support by Ru species [49]. Nevertheless, Ru/SnO_2_-Sp demonstrates a notably higher specific surface area in comparison with Ru/SnO_2_-Sh and Ru/SnO_2_-Rd, which would be favorable to its catalytic activity for VC oxidation.

### 3.3. Redox Behavior

**Figure 6:**
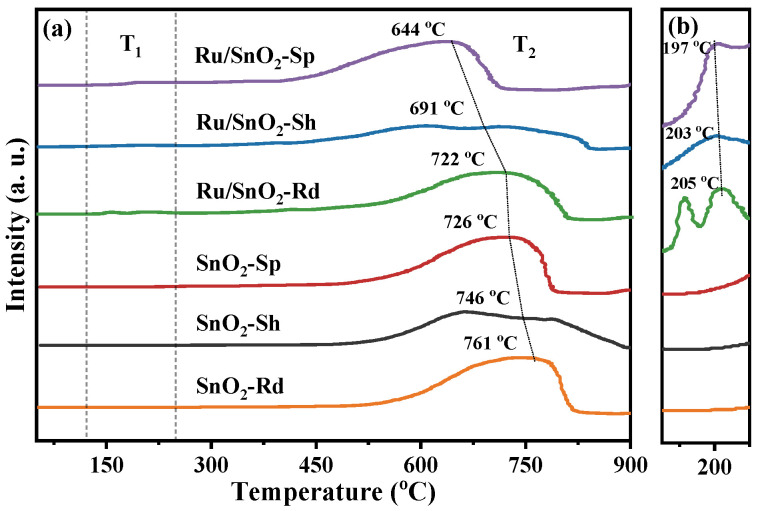
H_2_-TPR profiles of SnO_2_ supports and Ru/SnO_2_ catalysts. (**a**) Full temperature H_2_-TPR curves range from 100 to 900 °C; (**b**) Magnified view of the low temperature T_1_ region. The dashed lines indicate the variation in the maximum reduction temperature of each reduction peak.

On the basis of the H_2_-TPR profiles in Figure 6a, all of SnO_2_-Sp, SnO_2_-Sh and SnO_2_-Rd supports exhibit distinct reduction peaks at high temperatures of above 500 °C, typically related to the reduction of Sn^4+^ into metallic Sn along with the dissociation of lattice oxygen from SnO_2_ [38,50]. Specifically, the reduction temperatures of peaks maximum over SnO_2_-Sp, SnO_2_-Sh and SnO_2_-Rd supports are 726, 746, and 761 °C, respectively. Among them, SnO_2_-Sp presents the lowest reduction temperature of peaks’ maximum, indicating that its lattice oxygen is more easily reducible and more active compared to the other two supports. This indirectly demonstrates more oxygen defects are available in the SnO_2_-Sp support. In addition, all SnO_2_ supports exhibit minimal hydrogen consumption at the low-temperature stage without any observable reduction peaks.

As shown in Figure 6a,b, the H_2_-TPR profiles of all Ru/SnO_2_ catalysts can be categorized into two counterparts at the low-temperature stage (T_1_) and the high-temperature stage (T_2_), respectively. During T_1_, the reduction of surface active oxygen species mainly occurs concurrently with the transformation of trace Ru^n+^ into Ru^0^ on the surface [47,50]. Herein, Ru^n+^ represents a mixed valence state of various oxidized ruthenium species. During T_2_, the reduction of lattice oxygen in the SnO_2_ support primarily takes place. At T_1_, the Ru/SnO_2_-Sp catalyst has the lowest reduction temperature of peak maximum as an indicator of its highest low-temperature reducibility. This finding may be ascribed to the unique spherical structure of the SnO_2_-Sp support, which provides a better dispersion environment for Ru species, allows Ru^n+^ to be more fully exposed on the catalyst surface, and thus facilitates its reduction with hydrogen. At T_2_, the reduction temperatures of peaks maximum over all Ru/SnO_2_ catalysts are relatively lower than those of their corresponding SnO_2_ supports. This finding evidently indicates that the introduction of Ru obviously enhances the reducible activity of lattice oxygen in SnO_2_ supports. As an active metal component, the surface-dispersed Ru emerges with a strong interaction with the SnO_2_ support, which can alter the surface electronic structure and chemical properties of the SnO_2_ support and enable lattice oxygen to be more reducible [49]. Among them, the reduction peak over Ru/SnO_2_-Sp at T_2_ significantly shifts towards the low-temperature orientation in contrast to the other two catalysts, giving the minimum reduction temperature at the peak maximum. This phenomenon aligns with the reduction behavior of the independent SnO_2_ supports and further proves the highest lattice oxygen reducibility over Ru/SnO_2_-Sp. The highly active lattice oxygen promotes the interaction between Ru and the SnO_2_ support, facilitates the formation of more stable active centers, and thereby is favorable for the catalytic oxidation reaction. Overall, the reducibility of the Ru/SnO_2_ catalysts follows a descending order of Ru/SnO_2_-Sp > Ru/SnO_2_-Sh > Ru/SnO_2_-Rd. Table 2 lists the maximum reduction temperature and hydrogen uptake amount for each characteristic peak over both SnO_2_ supports and Ru/SnO_2_ catalysts at T_1_. One can note that the hydrogen consumption corresponding to the reduction of Ru^n+^ to Ru^0^ follows the decreasing trend of Ru/SnO_2_-Sp (0.118 mmol g^−1^) > Ru/SnO_2_-Sh (0.066 mmol g^−1^) > Ru/SnO_2_-Rd (0.055 mmol g^−1^), which implies that the Ru/SnO_2_-Sp catalyst possesses stronger oxygen activation capability [51]. Comprehensively, it can be speculated that the morphology of SnO_2_ supports has an important impact on the dispersion status, the valence state distribution and the low-temperature reducibility of Ru species. And the Ru/SnO_2_-Sp catalyst exhibits the best low-temperature reducibility.

**Figure 7:**
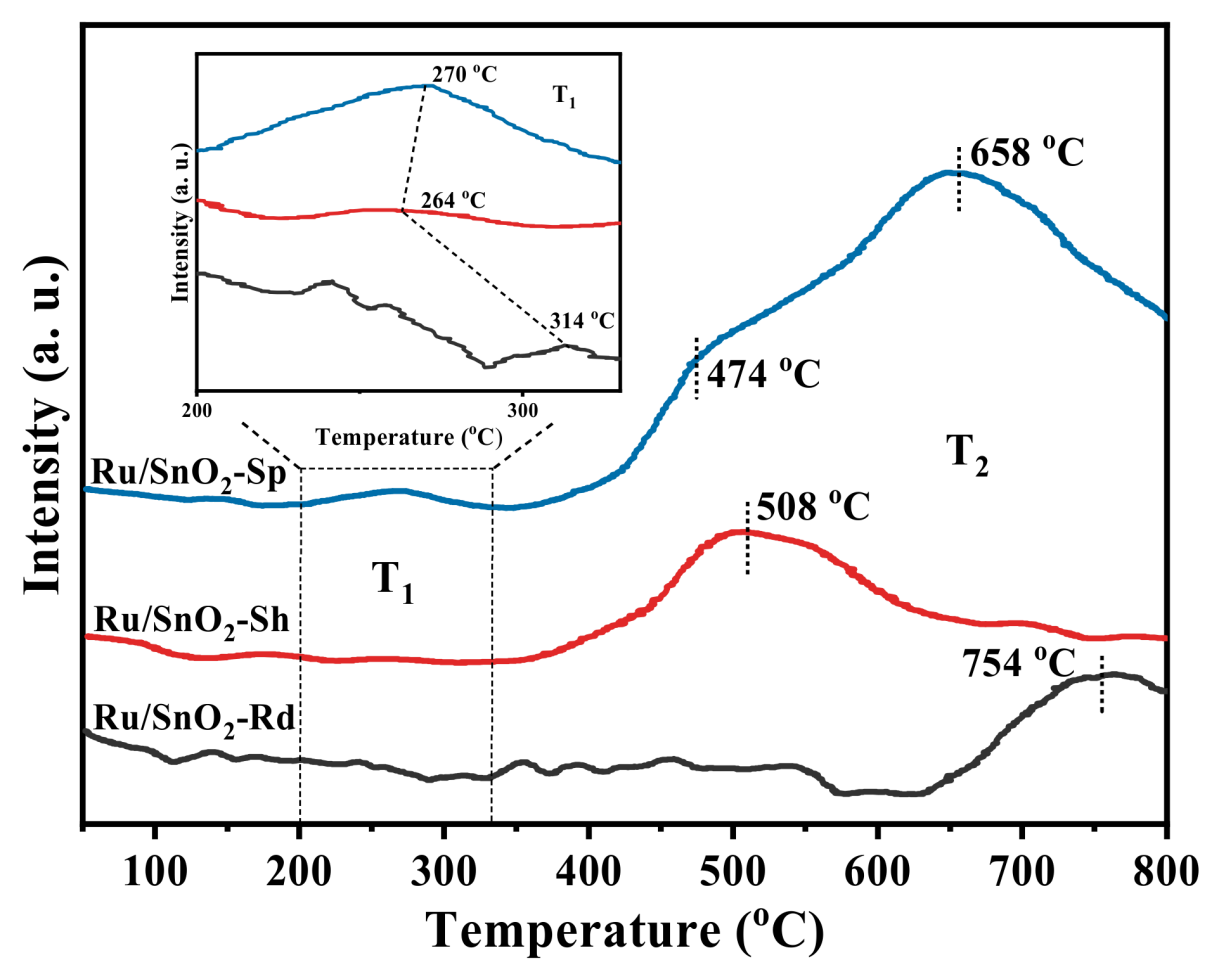
O_2_-TPD profiles of Ru/SnO_2_ catalysts.

As shown in Figure 7, one can find that all Ru/SnO_2_ catalysts exhibit two oxygen desorption peaks at the low-temperature range from 200 to 320 °C and the high-temperature range from 400 to 800 °C. There appears a weak oxygen desorption signal below 250 °C, which typically results from the desorption of surface-adsorbed oxygen. As the crucial active species in catalytic VOC oxidation, a greater quantity of surface-adsorbed oxygen is conducive to the catalytic activity improvement [52,53]. With the temperature rise, there appears a prominent desorption peak in a wide range of 400–800 °C, which can be ascribed to the desorption of the surface lattice oxygen. Based on the peak area comparison of each catalyst in Figure 7, it is evident that the peak areas follow a descending order of Ru/SnO_2_-Sp > Ru/SnO_2_-Sh > Ru/SnO_2_-Rd, which reflects their variable oxygen desorption quantities. Among them, Ru/SnO_2_-Sp exhibits the lowest temperature (ca. 474 °C) and the strongest signal intensity corresponding to surface lattice oxygen desorption. This phenomenon indicates that more oxygen species migrate from the lattice surface to adjacent vacancies over the Ru/SnO_2_-Sp catalyst with the increasing temperature, which is in close correspondence with the high-temperature reduction peaks in the previous H_2_-TPR profiles. The substantial migration of lattice oxygen can provide more surface active sites, which will effectively promote catalytic VC oxidation.

### 3.4. Elemental Analysis

**Figure 8:**
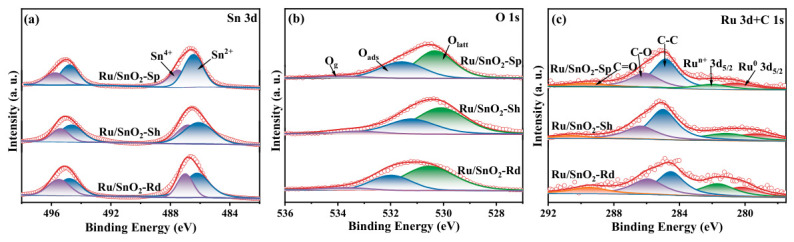
XPS spectra of (**a**) Sn 3d, (**b**) O 1s and (**c**) Ru 3d + C 1s over Ru/SnO_2_ catalysts.

Figure 8a displays the XPS spectra of Sn 3d over the Ru/SnO_2_ catalysts, which shows two characteristic peaks at binding energies of 487.5 and 486.4 eV typically corresponding to Sn^4+^ and Sn^2+^ cations, respectively [54]. Previous research demonstrated that Sn^4+^ is the primary species in the SnO_2_ support [38]. As noticed in Table 3, the multiple valence states consisting of Sn^2+^ and Sn^4+^ appear over the Ru/SnO_2_ catalysts, showing variable relative ratios of Sn^2+^/Sn^4+^ in a decreasing order of Ru/SnO_2_-Sp (1.60) > Ru/SnO_2_-Sh (1.40) > Ru/SnO_2_-Rd (1.07). This phenomenon can be ascribed to the strong electron-attracting effect of oxygen vacancies on adjacent Sn^4+^ ions. In order to maintain a charge neutrality, a certain amount of Sn^4+^ cations would spontaneously transform into Sn^2+^ cations [54]. It has been demonstrated that the higher ratio of Sn^2+^/Sn^4+^ implies the greater appearance of oxygen vacancies on the catalyst surface. Consequently, the Ru/SnO_2_-Sp catalyst shows the generation of a greater number of oxygen vacancies due to its higher relative proportion of Sn^2+^/Sn^4+^. This finding is in close accordance with the outcomes derived from the O_2_-TPD analysis.

In Figure 8b, the O 1s spectra over Ru/SnO_2_ catalysts can be resolved into three components with binding energies at 533.6, 531.6 and 530.3 eV, characteristically corresponding to the surface hydroxyl and water groups (O_g_), surface-adsorbed oxygen species (O_ads_) and lattice oxygen species (O_latt_), respectively [12]. As noticed in Table 3, one can find that the Ru/SnO_2_-Sp catalyst exhibits the highest O_ads_/O_latt_ (0.65), indicating that its surface is rich in more oxygen species. This suggests that the morphology of the SnO_2_ support has an effect on the amount of active surface oxygen. Consequently, this has an influence on the oxidation activity of VC.

In Figure 8c, one can find that the peak with its center at 280.2 eV is attributed to metallic ruthenium (Ru^0^), and the peak at 281.6 eV corresponds to oxidized ruthenium (Ru^n+^), respectively [47]. Based on the quantitative analysis in Table 3, the Ru^0^/Ru^n+^ ratio of the catalysts ranks in the sequence Ru/SnO_2_-Rd (0.75) < Ru/SnO_2_-Sh (0.86) < Ru/SnO_2_-Sp (1.40). The Ru/SnO_2_-Sp exhibits the highest Ru^0^/Ru^n+^ ratio, corresponding to the highest surface loading of metallic Ru^0^ species.

### 3.5. Catalytic Performances

**Figure 9:**
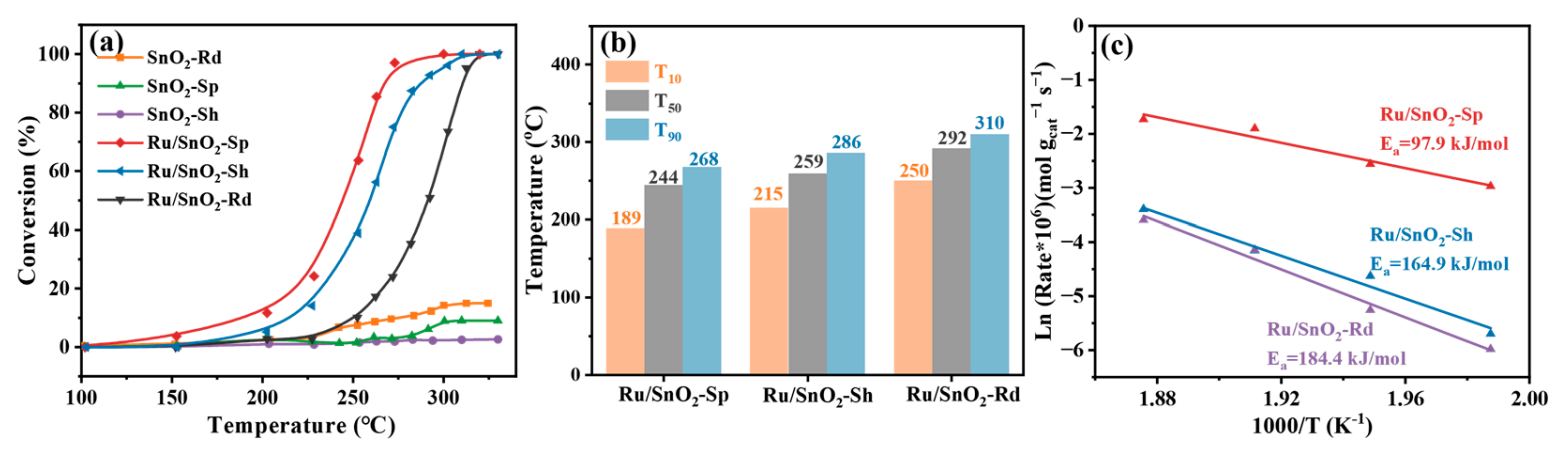
(**a**) Light-off curves as a function of reaction temperature, (**b**) the characteristic values of T_10_, T_50_ and T_90_, and (**c**) Arrhenius plots for VC oxidation over SnO_2_ supports and Ru/SnO_2_ catalysts.

Figure 9 presents the light-off curves of VC oxidation in relation to the reaction temperature. It also shows the characteristic conversion temperatures corresponding to 10%, 50%, and 90% VC conversion (denoted as T_10_, T_50_ and T_90_, respectively), along with the Arrhenius plots utilized for calculating the apparent activation energy (*E*_a_). As shown in Figure 9a, SnO_2_ supports exhibit negligible VC conversion across the whole tested temperature interval, suggesting their inferior catalytic performance. In contrast, Ru/SnO_2_ catalysts demonstrate significantly high catalytic activity. This proves that even a small content of Ru loading can substantially enhance the overall catalytic activity, which arises from the formation of Ru^0^/Ru^n+^ redox-active sites. Based on the light-off curves presented in Figure 9a, the catalytic performance of the Ru/SnO_2_ catalysts follows an ascending order of Ru/SnO_2_-Rd < Ru/SnO_2_-Sh < Ru/SnO_2_-Sp. Obviously, Ru/SnO_2_-Sp exhibits the optimum catalytic performance for VC oxidation with the same loading content of Ru as the other catalysts. Specifically, Ru/SnO_2_-Sp achieves the minimum T_10_, T_50_ and T_90_ values, which are 189, 244 and 268 °C, respectively (Figure 9b). For one thing, such outstanding catalytic activity is attributed to the largest specific surface area of the SnO_2_-Sp support, which promotes the surface dispersion of Ru species. For another, the SMSI, together with the plentiful surface-adsorbed oxygen and oxygen vacancies on the Ru/SnO_2_-Sp surface, creates advantageous conditions for the catalytic oxidation of VC.

Kinetic experiments were implemented under a strict kinetic regime in order to compare the inherent catalytic capabilities of the catalysts. Based on the Arrhenius plot, the reaction rate at 210 °C and the *E*_a_ of each catalyst in Figure 9c and Table 4, the VC conversion and reaction rates of the catalysts are consistent with their catalytic activities in Figure 9a, obeying the sequence of Ru/SnO_2_-Rd < Ru/SnO_2_-Sh < Ru/SnO_2_-Sp. Correspondingly, linear Arrhenius plots displayed in Figure 9c were used to acquire apparent activation energies, which rank in the inverse order of Ru/SnO_2_-Rd (184.4 kJ mol^−1^) > Ru/SnO_2_-Sh (164.9 kJ mol^−1^) > Ru/SnO_2_-Sp (97.9 kJ mol^−1^). The turnover frequency (TOF) values normalized by surface-exposed Ru atoms for three catalysts are listed in Table 4. Ru/SnO_2_-Sp exhibits a remarkably higher TOF (3.01 × 10^−3^ s^−1^) than Ru/SnO_2_-Sh (0.26 × 10^−3^ s^−1^) and Ru/SnO_2_-Rd (0.11 × 10^−3^ s^−1^). The lower apparent activation energy, the higher reaction rate, and the larger TOF over Ru/SnO_2_-Sp confirm its superior catalytic behavior for catalytic VC oxidation. Moreover, the catalytic activity of the Ru/SnO_2_-Sp catalyst is compared with other Ru-based catalytic materials previously reported in the literature in terms of the T_50_ and T_90_ parameters. As shown in Table S1, it can be found that the Ru/SnO_2_-Sp catalyst exhibits significantly higher catalytic activity for VC oxidation with the lowest T_50_ and T_90_ values under the similar reaction conditions in comparison with other catalysts, thus strongly demonstrating its competitiveness and application potentiality. Combined with the above characterization results and kinetic tests, the SnO_2_-Sp support possesses a larger specific surface area and developed pore structure, which effectively restrains the agglomeration of Ru nanoparticles and exposes abundant active sites. Meanwhile, the strong metal–support interaction (SMSI) between Ru and SnO_2_ induces the generation of abundant oxygen vacancies, remarkably increasing the O_ads_/O_latt_ ratio over the Ru/SnO_2_-Sp catalyst. The enriched surface adsorbed oxygen greatly reduces the activation energy required for the reaction. In addition, metallic Ru^0^ species on this catalyst act as the core active sites for activating the C−Cl bond of VC and deeply oxidizing reaction intermediates. The synergistic effect of the above structural advantages and electronic defect regulation significantly reduces the apparent activation energy for VC oxidation, endowing Ru/SnO_2_-Sp with superior low-temperature catalytic activity compared with catalysts reported in previous literature.

**Figure 10:**
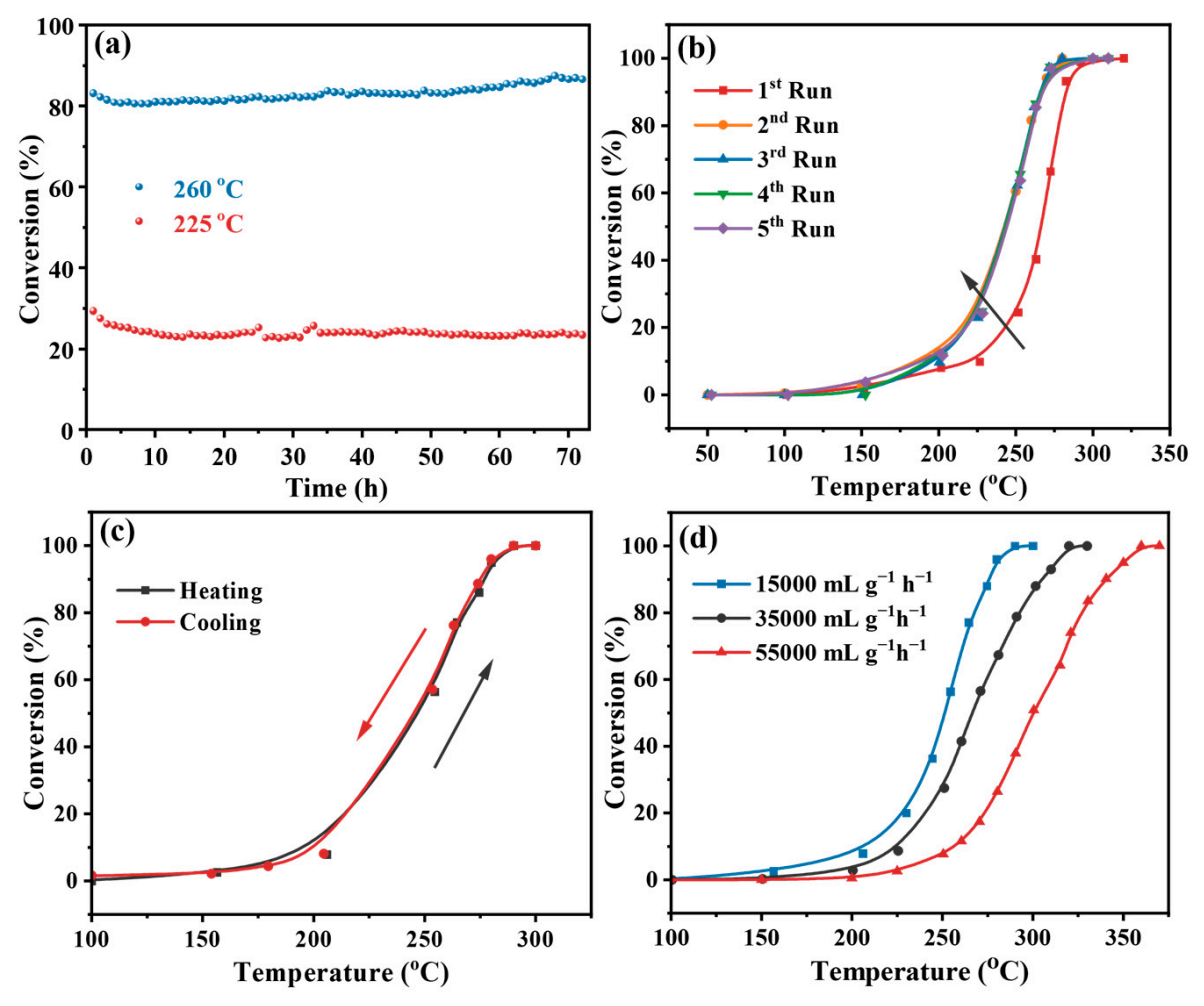
(**a**) Continuous 72 h aging performance under 225 and 260 °C reaction temperatures, (**b**) five-cycle repeated tests, (**c**) a comparative analysis under heating and cooling conditions, and (**d**) under variable space velocities over the Ru/SnO_2_-Sp catalyst.

During catalytic VC oxidation, the adsorbed chlorine species on the catalyst surface preferentially occupy active sites, triggering irreversible deactivation. In this study, a continuous catalytic test for 72 h was conducted over the Ru/SnO_2_-Sp catalyst at 225 and 260 °C to evaluate its long-term reaction durability. As shown in Figure 10a, Ru/SnO_2_-Sp exhibits stable VC conversion at approximately 25 and 80% without any significant decline throughout the entire testing period, indicating its excellent long-term durability. To further evaluate the catalytic durability, light-off measurements were performed over the fresh catalyst and the spent catalyst recovered after the 72 h long-term stability test, and the corresponding curves are displayed in Figure S2. It can be observed that the T_10_, T_50_ and T_90_ values of the spent catalyst nearly overlap with those of the fresh catalyst without obvious activity deterioration. This result verifies that the Ru/SnO_2_-Sp catalyst maintains outstanding catalytic activity after a long period of reaction, which further confirms its excellent thermal and catalytic stability.

In order to evaluate the multiple-cycle stability of the Ru/SnO_2_-Sp catalyst, five consecutive catalytic runs of VC oxidation were carried out. In Figure 10b, the curves of VC conversion in the second run significantly shift towards the low-temperature direction after the first run, which implies that the catalytic activity of Ru/SnO_2_-Sp further enhances rather than decreases after its first usage in the experiment. This phenomenon may be related to the valence state redistribution of Ru species after the first catalytic run. However, the curves of VC conversion in the third to the fifth runs are essentially overlapped with that in the second run, which suggests the non-occurrence of catalyst deactivation and the good cyclic stability of this catalyst. To further verify the valence evolution of Ru species during multiple cycling catalytic reactions, XPS measurements were performed over the Ru/SnO_2_-Sp catalysts after the first and five consecutive runs, which were denoted as 1-cycle and 5-cycle Ru/SnO_2_-Sp, respectively. In Figure S3 and Table S2, the fresh catalyst possesses a Ru^0^/Ru^n+^ molar ratio of 1.40 and an O_ads_/O_latt_ ratio of 0.65. After one catalytic cycle, these two values shift to 0.98 and 0.72, respectively. It proves that the reaction process can contribute to the generation of more Ru^n+^ species and abundant adsorbed active oxygen on the catalyst surface. Correspondingly, the T_90_ of the catalyst decreases from 282 °C to 265 °C, which directly reveals that the increased oxygen vacancies and enriched active oxygen effectively reduce the apparent activation energy for vinyl chloride oxidation. After five cycles, the Ru^0^/Ru^n+^ and O_ads_/O_latt_ molar ratios of the catalyst stabilize at 0.95 and 0.71, respectively. The corresponding T_90_ only slightly rises to 268 °C, which is basically consistent with the parameters of the catalyst after one cycle. This demonstrates that the valence states of Ru species and oxygen distribution remain basically unchanged after five consecutive reactions. Accordingly, the catalyst can still maintain high catalytic activity toward VC oxidation after multiple cycles.

Figure 10c compares the catalytic activities for VC oxidation over Ru/SnO_2_-Sp during the heating and cooling processes. The overlapped light-off curves indicate that VC conversion remains nearly unchanged at the same temperature in both processes. This phenomenon can be owing to the stable microstructure and surface properties of the catalyst; thus, the quantity and nature of active sites don’t change with temperature variations, which ensures the consistent catalytic performance and reflects a stable surface interaction between the reactant molecules and the catalytic active sites.

Through adjusting the mass of the loaded catalyst, the catalytic activities of the Ru/SnO_2_-Sp catalyst for VC oxidation under different space velocities were assessed. In Figure 10d, as the space velocity increases from 15,000 to 55,000 mL g^−1^ h^−1^, the light-off curves of VC conversion gradually shift towards the high-temperature region, indicating the occurrence of catalytic activity decline at higher space velocity. This finding is mainly owing to the shortened contact time between reactant molecules and surface active sites at high space velocity. Nevertheless, the Ru/SnO_2_-Sp catalyst can achieve complete VC conversion at 360 °C even under a high space velocity of 55,000 mL g^−1^ h^−1^, which confirms its good operational applicability across a broad spectrum of reaction conditions.

**Figure 11:**
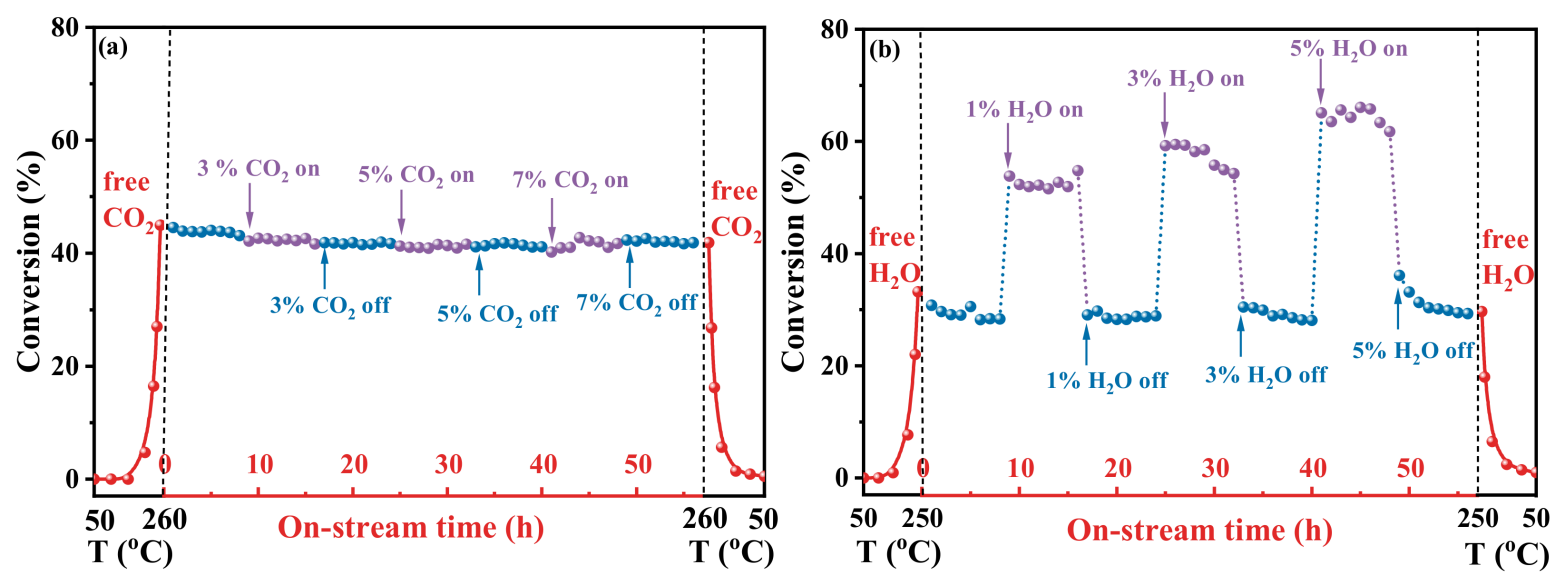
Variations of VC conversion with temperature and on-stream time over Ru/SnO_2_-Sp in (**a**) CO_2_ and (**b**) humid conditions.

The resistant ability of the Ru/SnO_2_-Sp catalyst towards CO_2_ and water in the reaction was extensively investigated under variable CO_2_-containing and humid conditions, and the relevant experiments were conducted at 260 and 250 °C, respectively. As seen in Figure 11a, 45% of VC conversion stably maintains with the increasing addition of CO_2_ into the reactant feed (from 3 vol.% to 7 vol.%), which indicates its excellent resistant ability towards CO_2_. This finding can be explained by the fact that the higher adsorption energy of VC molecules on the active sites of Ru/SnO_2_-Sp, comparable to CO_2_ molecules, makes them much easier to occupy the active centers, thus inhibiting the competitive adsorption of CO_2_ on active sites. In Figure 11b, one can note that Ru/SnO_2_-Sp exhibits stable VC conversion of ca. 30% at 250 °C under water-free conditions during the initial 8 h test. Interestingly, after introducing 1 vol.%, 3 vol.% and 5 vol.% water vapor into the reaction gas, the conversion rate of VC increased gradually to 50%, 60% and 65%. Water vapor participates in the catalytic reaction and promotes the removal of chlorine species adsorbed on the catalyst surface as HCl, which exposes more active sites on the Ru/SnO_2_-Sp, thus enhancing the conversion efficiency [55,56,57]. Only water vapor and CO_2_ were introduced to simulate industrial flue gas in this work, while other coexisting components were not considered. More complex mixed gas conditions will be adopted in subsequent studies.

### 3.6. Structure–Activity Relationship

In this work, Ru/SnO_2_-Sp possesses a larger specific surface area and superior pore structure compared with Ru/SnO_2_-Sh and Ru/SnO_2_-Rd. Such favorable morphological features enable the uniform dispersion of Ru nanoparticles and expose more accessible active sites on the catalyst, fully demonstrating the promotional effect of morphology on catalytic oxidation activity.

H_2_-TPR and O_2_-TPD characterizations verify that Ru loading markedly improves the redox capacity of the SnO_2_-Sp support. Among all catalysts, Ru/SnO_2_-Sp delivers the optimal low-temperature reducibility and the richest surface reactive oxygen species. Combined with kinetic measurements, the outstanding redox properties of Ru/SnO_2_-Sp greatly reduce the apparent activation energy for VC oxidation and fundamentally accelerate the catalytic reaction rate. The above characterization results solidly confirm the intrinsic correlation between redox performance and catalytic activity. Furthermore, XPS results further reveal that Ru/SnO_2_-Sp exhibits the highest O_ads_/O_latt_ ratio (0.65) and Ru^0^/Ru^n+^ ratio (1.40). A higher Ru^0^/Ru^n+^ ratio indicates that the surface of Ru/SnO_2_-Sp is rich in highly active metallic Ru^0^ sites, which facilitate the rapid activation and degradation of VC and reaction intermediates. The elevated O_ads_/O_latt_ ratio demonstrates abundant oxygen vacancy defects on the catalyst, which effectively cut down the activation energy required for VC conversion.

Highly dispersed Ru nanoparticles, sufficient oxygen vacancies and excellent low-temperature redox performance generate a prominent synergistic effect. Collectively, these factors lower the activation energy of vinyl chloride oxidation and endow Ru/SnO_2_-Sp with the optimal catalytic oxidation performance.

### 3.7. Reaction Mechanism

**Figure 12:**
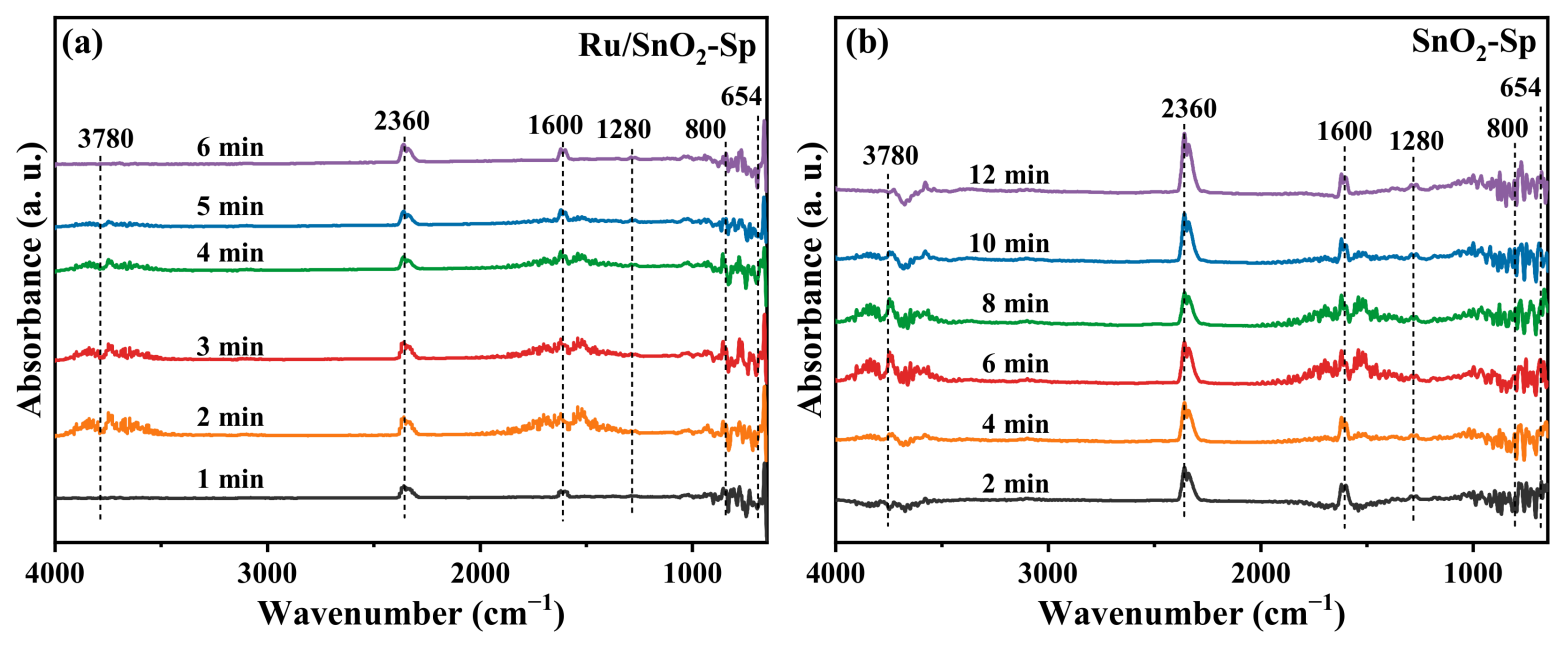
In situ DRIFTs data of (**a**,**b**) VC adsorption at 50 °C over Ru/SnO_2_-Sp and SnO_2_-Sp.

In situ DRIFTs was conducted to systematically investigate VC adsorption behaviors, surface intermediate species, reaction pathways and the intrinsic reaction mechanism over SnO_2_-Sp and Ru/SnO_2_-Sp, and the assignments of infrared absorption peaks are listed in Table S3. Figure 12a,b illustrate the temporal evolution of VC adsorption behaviors on the catalyst surface at a constant temperature of 50 °C. For the Ru/SnO_2_-Sp catalyst, the stretching vibrations corresponding to VC molecules, namely ν_as_(C–Cl) at 654 and 800 cm^−1^, ν_as_(CH_2_) at 1280 cm^−1^ and ν(C=C) at 1600 cm^−1^ gradually intensify and reach a stable intensity at 6 min, indicating VC adsorption saturation [3,58]. For the SnO_2_-Sp support, it took 12 min to reach VC adsorption–desorption equilibrium. The loading of Ru introduces abundant adsorptive active sites on the catalyst surface, thereby enhancing the adsorption efficiency for VC molecules. In contrast, the bare SnO_2_-Sp support only possesses weak physisorption sites, which accounts for its relatively slower adsorption equilibrium rate.

**Figure 13:**
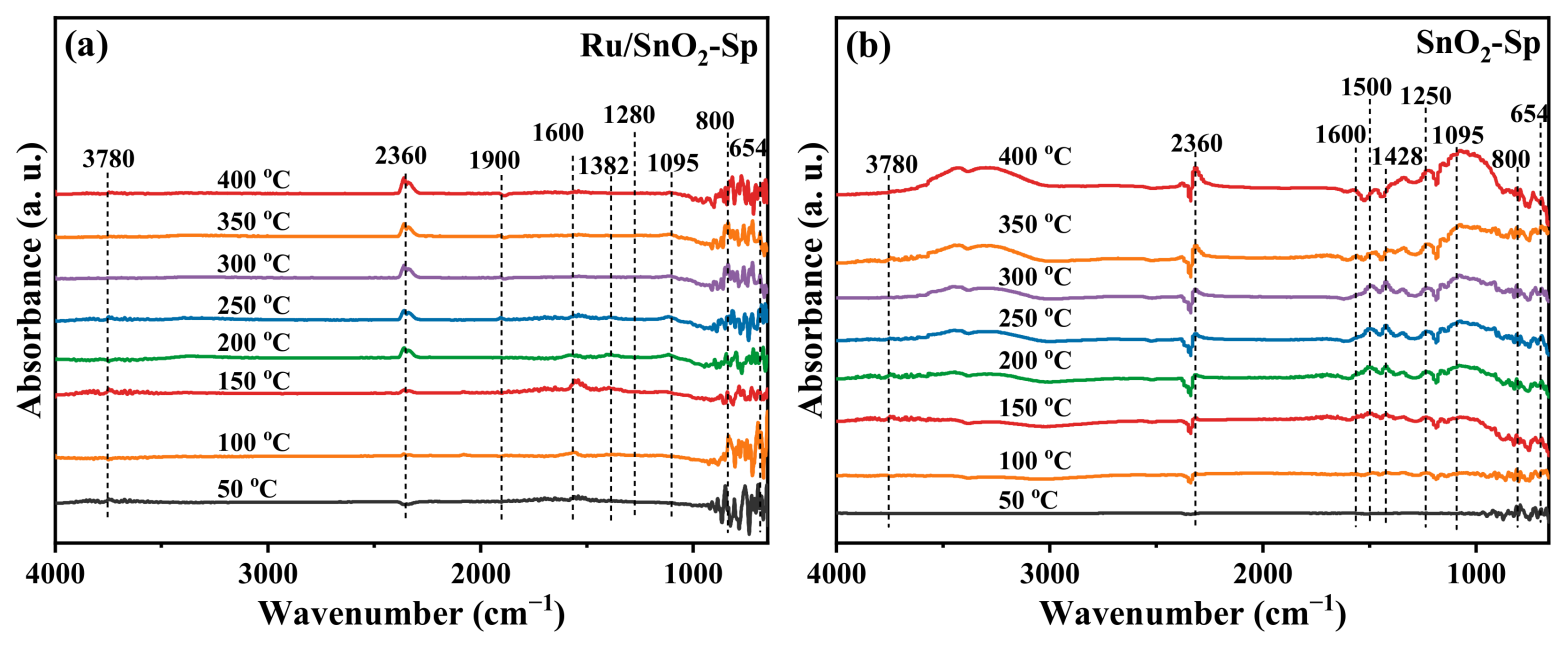
In situ DRIFTs data of (**a**,**b**) VC oxidation from 50 to 400 °C over Ru/SnO_2_-Sp and SnO_2_-Sp.

Figure 13a,b present the in situ DRIFTs data for VC oxidation over Ru/SnO_2_-Sp and SnO_2_-Sp from 50 °C to 400 °C. For Ru/SnO_2_-Sp, the peak intensity assigned to adsorbed VC at 654, 800, 1280 and 1600 cm^−1^ gradually weakens as temperature rises. Even at 400 °C, these peaks can still be clearly identified, revealing persistent adsorbed chlorinated organics over the catalyst. In addition, relevant research on VC oxidation over Ru/SnO_2_ systems has detected five typical chlorinated byproducts under identical reaction conditions, namely 1,1,2-trichloroethane, trans-1,2-dichloroethylene, dichloromethane, chloroform and carbon tetrachloride [12]. Notably, the IR peak at 1382 cm^−1^ corresponding to enol species initially emerges at 150 °C, and its intensity gradually increases with temperature rise and subsequently disappears at 300 °C. This mirrors the formation and decomposition processes of enol intermediates [12,59]. Between 200 and 400 °C, there appear the IR peaks at 1900 and 1095 cm^−1^, typically corresponding to carboxylic acid species, which reveals the further oxidation of enol species into carboxylic acid on the catalyst surface [60,61]. In the higher temperature from 250 to 400 °C, the IR peak at 2360 cm^−1^ exhibits progressive intensity enhancement as the temperature rises, indicating the accelerated CO_2_ formation kinetics [62]. Meanwhile, a characteristic IR peak of adsorbed water emerges at 3780 cm^−1^, confirming the generation of H_2_O during the reaction process. For the SnO_2_-Sp support, only IR peaks of VC at 800, 654, 1280 and 1600 cm^−1^ are observed in the temperature range from 50 to 200 °C, with barely any IR peaks of other organic intermediates detected. At 150 °C, the IR peaks corresponding to enol species at 1428 and 1500 cm^−1^, and carboxylic acid at 1095 cm^−1^ are gradually observed, showing a weak CO_2_ adsorption signal ultimately. These phenomena confirm the extremely poor catalytic activity of the bare SnO_2_-Sp support for VC oxidation at low temperature, and VC molecules and these organic intermediates cannot be completely oxidized.

**Figure 14:**
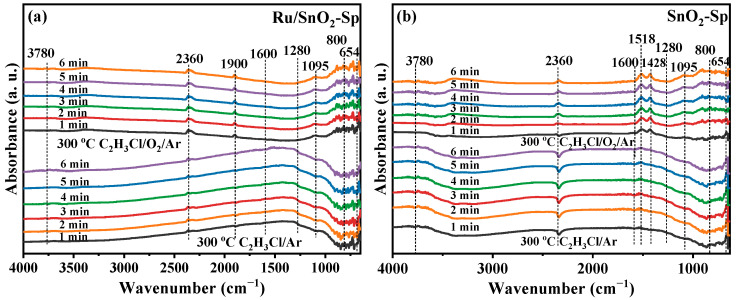
In situ DRIFTs data of (**a**,**b**) VC oxidation at 300 °C over Ru/SnO_2_-Sp and SnO_2_-Sp.

Figure 14a,b display the in situ DRIFTs plotted with respect to the operational duration at 300 °C. These IR spectra were gathered in both oxygen-absent and oxygen-present atmospheres for Ru/SnO_2_-Sp and SnO_2_-Sp. For Ru/SnO_2_-Sp, the peak intensity of VC characteristic bands at 654, 800, 1280 and 1600 cm^−1^ on Ru/SnO_2_-Sp gradually increases and reaches stable values within 5 min under an oxygen-absent atmosphere. Once oxygen is fed into the reaction system, these characteristic bands progressively diminish in intensity. Additionally, the IR peaks corresponding to carboxylic acid species appear at 1095 and 1900 cm^−1^. This phenomenon indicates the participation of surface active oxygen species in the reaction, thus promoting the VC conversion into organic intermediates containing carboxylic acid groups. Notably, the IR peak of CO_2_ at 2360 cm^−1^ exhibits a remarkable intensity enhancement, which is due to the efficient oxygen activation and oxidation of organic intermediates into CO_2_ by Ru/SnO_2_-Sp under oxygen-present atmospheres. For the bare SnO_2_-Sp support, the rising intensities of VC bands also correspond to the adsorption of VC molecules. However, IR peaks attributable to enol species appear at 1428 and 1518 cm^−1^ upon the introduction of oxygen. Considering the weak IR peak of CO_2_ at 2360 cm^−1^, it can be inferred that the organic intermediates are difficult to be completely decomposed into CO_2_ on account of the extremely low catalytic activity of SnO_2_-Sp.

**Scheme 1:**
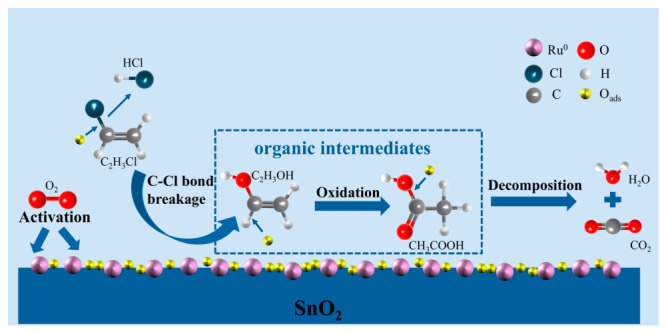
Speculated catalytic mechanism and reaction routes for VC oxidation catalyzed by Ru/SnO_2_-Sp under oxidative conditions.

A reasonable reaction mechanism is put forward to clarify the catalytic conversion routes of VC molecules and key organic intermediates over the Ru/SnO_2_-Sp surface. As illustrated in Scheme 1, gaseous oxygen molecules in the reactant feed can be catalytically activated on the catalyst. This activation proceeds through oxygen vacancies located at the distinct interface where metallic Ru^0^ contacts the SnO_2_-Sp support. As a result of this activation, oxygen molecules are converted into surface oxygen species (O_ads_), which are widely acknowledged to exhibit high reactivity in catalytic reactions. VC molecules experience chemisorption on the surface of the catalyst, rendering them more chemically reactive and thermodynamically unstable. Subsequently, the C–Cl bonds of adsorbed VC are attacked by active O_ads_, triggering C–Cl bond cleavage and releasing HCl. Side reactions may simultaneously generate chlorinated byproducts, including 1,1,2-trichloroethane, trans-1,2-dichloroethene, dichloromethane, chloroform and carbon tetrachloride. Meanwhile, VC is initially oxidized into enol intermediates; the enol intermediates are further oxidized to carboxylic acid intermediates by O_ads_. Eventually, given the innate instability of carboxylic acid intermediates, they are easily decomposed into CO_2_ and H_2_O. Throughout the catalytic cycle, surface O_ads_ species dominate the VC oxidation process over Ru/SnO_2_-Sp. The overall reaction pathway is consistent with the Mars–van Krevelen mechanism, which is in line with the catalytic oxidation behavior of most VOCs over supported noble metal catalysts [63,64].

## 4. Conclusions

Three types of Ru/SnO_2_ catalysts with different morphologies were fabricated for VC catalytic oxidation, and the morphological feature of SnO_2_ supports strongly governs their catalytic performances. The nanosphere catalyst of Ru/SnO_2_-Sp presents not only the optimum catalytic activity but also impressive long-term catalytic durability and cyclic stability as well as CO_2_/water resistance ability under complex industrial conditions. The structure–reactivity relationship indicates the predominant roles of metallic Ru^0^ and O_ads_ from Ru/SnO_2_-Sp interface interaction in catalytic VC oxidation. The in situ DRIFTs evidenced that the enol species, original from C-Cl bond cleavage and initial activation of VC molecules, and the carboxylic acid species, from the subsequent oxidation of enol, are both identified as important organic intermediates. This work provides fundamental guidance for the development of Ru-based catalysts and understanding the reaction mechanism for CVOCs’ oxidation.

## Figures and Tables

**Table 1:** Porosity parameters by nitrogen sorption measurements over SnO_2_ supports and Ru/SnO_2_ catalysts.

Catalysts	Specific Surface Area (m^2^ g^−1^)	Total Pore Volume (cm^3^ g^−1^)	Pore Diameter (nm)
SnO_2_-Sp	30.4	0.20	8.12
SnO_2_-Sh	13.8	0.10	3.31
SnO_2_-Rd	6.3	0.04	3.31
Ru/SnO_2_-Sp	20.2	0.09	3.71
Ru/SnO_2_-Sh	17.9	0.09	3.71
Ru/SnO_2_-Rd	8.6	0.07	3.13

**Table 2:** Quantitative data obtained from H_2_-TPR profiles over SnO_2_ supports and Ru/SnO_2_ catalysts.

Catalysts	Maximum Reduction Temperature (°C)		H_2_ Uptakes (mmol g^−1^)
	T_1_	T_2_	
SnO_2_-Sp	-	726	-
SnO_2_-Sh	-	746	-
SnO_2_-Rd	-	761	-
Ru/SnO_2_-Sp	197	644	0.118
Ru/SnO_2_-Sh	203	691	0.066
Ru/SnO_2_-Rd	205	717	0.055

**Table 3:** Accurate O 1s binding energies and quantitative fitting results obtained from XPS peak deconvolution of Ru/SnO_2_ catalysts.

Catalysts	O 1s			Sn^2+^/Sn^4+^	O_ads_/O_latt_	Ru^0^/Ru^n+^
	O_ads_	O_latt_	O_g_			
Ru/SnO_2_-Sp	531.6	530.3	533.8	1.60	0.65	1.40
Ru/SnO_2_-Sh	531.2	530.1	533.6	1.40	0.59	0.86
Ru/SnO_2_-Rd	531.9	530.6	533.6	1.07	0.43	0.75

**Table 4:** Results of kinetic investigations for VC oxidation over Ru/SnO_2_ catalysts.

Catalysts	Conversion (%) ^a^	Reaction Rate (×10^−9^ mol g_cat_^−1^ s^−1^) ^a^	TOF (×10^−3^ s^−1^)	*E_a_* (kJ mol^−1^)	R^2^	MRD (%)
Ru/SnO_2_-Sp	4.23	52.44	3.01	97.9	0.96	4.20
Ru/SnO_2_-Sh	0.20	2.47	0.26	164.9	0.98	2.12
Ru/SnO_2_-Rd	0.11	1.31	0.11	184.4	0.98	2.21

^a^ Determined at 210 °C.

## Data Availability

The original contributions presented in this study are included in the article/Supplementary Materials. Further inquiries can be directed to the corresponding author.

## Supplementary Materials

The following supporting information can be downloaded at: https://www.mdpi.com/article/10.3390/nano16140850/s1. 1. Experimental and characterization details. 2. Reaction kinetics measurement. 3. Supplementary figures and tables. Figure S1: N_2_ adsorption-desorption isotherms of SnO_2_ supports. Figure S2. (a) Light-off curves as a function of reaction temperature and (b) the characteristic values of T_10_, T_50_, T_90_ over fresh Ru/SnO_2_-Sp catalyst and spent catalyst after 72 h long-term stability test. Figure S3. (a) Ru 3d + C 1s, (b) O 1s and (c) Sn 3d XPS spectra of the fresh, 1-cycle and 5-cycle Ru/SnO_2_ catalysts. Table S1. Catalytic activity comparison of the catalyst with those reported in the literatures. Table S2. The detailed binding energies of O 1s and the quantitative results from XPS spectra deconvolution over the fresh, 1-cycle and 5-cycle Ru/SnO_2_-Sp catalysts. Table S3. Frequencies of functional groups present on the SnO_2_-Sp and Ru/SnO_2_-Sp catalysts analyzed by DRIFTs. References [12,15,47,61,65,66,67,68] are cited in the Supplementary Materials.
