# Supplementary material for: Morphology-Dependent SnO2 Supported Ru Catalysts for Catalytic Oxidation of Vinyl Chloride Emission
Source: Nanomaterials (Basel). 2026 Jul 10;16(14):850. doi: 10.3390/nano16140850 (PMC13414581; doi:10.3390/nano16140850)
Supplement: Supplementary file 1 [file nanomaterials-16-00850-s001.zip › nanomaterials-4409809-supplementary.pdf]

Supplementary material

Morphology-Dependent SnO_2 Supported Ru Catalysts for Catalytic Oxidation of Vinyl Chloride Emission

Hongyu Cui [1], Mingju Wang [1], Maosheng Zhou [1], Tianqi Cao [1], Junyi Liu [1] and Chuanhui Zhang [1,2,*]

[1] Institute of Materials for Energy and Environment, College of Materials Science and Engineering, Qingdao University, Qingdao 266071, China
[2] Faculty of Chemical Engineering and Energy Technology, Shanghai Institute of Technology, Shanghai 201418, China
* Correspondence: zhangch@qdu.edu.cn

1. Experimental and characterization details

1.1. Experimental details

HCl is only used to regulate the morphology of SnO_2-Sp support. Chloride ions can be completely removed after multiple centrifugal water washing and calcination at 500 °C, and thus will not interfere with the subsequent Ru loading process. All three supports adopt the same post-treatment procedure, and impurities introduced by different hydrothermal conditions can be eliminated via high-temperature calcination. Variations in hydrothermal temperature, holding time and additives only alter the crystal and pore morphology of the supports, which are the core research variables of this work.

1.2. Characterization details

Powder X-ray diffraction (XRD) patterns were recorded on a Rigaku Ultima IV diffractometer with Cu Kα (λ = 0.154184 nm) radiation at 40 kV and 40 mA. Field emission scanning electron microscopy (FE-SEM) and transmission electron microscopy (TEM) were collected on the JSM-7800F and JEOL JEM-2100 Plus microscope, respectively. Nitrogen adsorption-desorption isotherms were first degassed at 200 °C for 10 h, and then tested by Quantachrome Autosorb-IQ3 sorptometer. Nitrogen adsorption-desorption isotherms were performed on a Quantachrome Autosorb-iQ3 sorptometer. Prior to the measurement, each catalyst was pretreated at 180 °C for 3 h under degassing conditions. Temperature-programmed reduction of hydrogen (H_2-TPR) was conducted on a Quantachrome Chembet Pulsar analyzer, the H_2-TPR profiles were collected in the range of 50-900 ^oC under 5% H_2/Ar flow after a pretreatment at 300 °C for 1 h under argon atmosphere. Temperature-programmed desorption of oxygen (O_2-TPD) was conducted on a Quantachrome Chembet Pulsar analyzer. The catalyst powder was pretreated at 200 °C for 30 min and naturally cooled to 50 °C under helium flow, and the desorption signal of oxygen was collected in the range of 50-900 °C at a heating rate of 10 °C min^{-1} under a helium flow. Raman spectra were collected in the anti-Stokes range of 50-1000 cm^{-1} using a Renishaw inVia Reflex Raman spectrometer (Renishaw PLC, Wotton-under-Edge, UK). X-ray photoelectron spectroscopy (XPS) spectra were conducted on PHI 5000 Versa Probe III spectrometer with a monochromatized Al Kα X-ray source (1486.6 eV). The binding energy (BE) was determined by utilizing C 1s of adventitious carbon (284.6 eV) as a reference.

In situ diffuse reflectance infrared Fourier transform spectroscopy (DRIFTs) was performed of the SnO_2-Sp and Ru/ SnO_2-Sp catalysts on a Nicolet iS50 spectrometer (Thermo Fisher SCIENTIFIC) with a mercury-cadmium-telluride (MCT) detector, and the DRIFTs cell with ZnSe windows is connected with a gas flow system. For VC adsorption analysis at 50 °C, the sample was pretreated at 50 °C in Ar atmosphere for 30 min. After the background spectra were recorded at the temperature, 1vol.% C_2H_3Cl/Ar with a flow rate of

20 mL min^{-1} was fed into the sample surface for adsorption, and in situ DRIFTs data of the sample was taken at different times. For VC adsorption and oxidation analysis at 300 °C, the sample was pretreated at 300 °C in Ar atmosphere for 30 min. After the background spectra were recorded at the temperature, Then, 1 vol.% C_2H_3Cl/Ar with a flow rate of 20 mL min^{-1} was fed into the sample surface for adsorption. After that, O_2 gas with a flow rate of 5 mL min^{-1} was continued to add to the reaction, and in situ DRIFTs data of the sample was taken at different times. For VC temperature-programmed oxidation, the sample was initially purged in the Ar stream at 400 °C for 30 min. After the background spectra were recorded at different temperatures, Ar gas was replaced by the gas mixture feed of 1vol.% C_2H_3Cl/Ar (20 mL min^{-1}) and O_2 (5 mL min^{-1}). The in situ DRIFTs data was recorded from 50 to 400 °C by temperature-programmed operation.

During the above experiments, background spectra were collected under pure Ar at the corresponding test temperature before gas introduction for baseline correction, which eliminates baseline drift originating from light scattering of catalyst powder and high-temperature thermal radiation.

2. Reaction kinetics measurement

To obtain the intrinsic kinetic parameters and eliminate the mass transfer interference, the catalyst dosage was adjusted to 30 mg for the kinetic tests, which was different from the catalyst amount used in the catalytic activity evaluation. High gas hourly space velocity (GHSV) was adopted to ensure that VC conversion was controlled below 10%, which satisfied the fundamental requirement for kinetics study.

The reaction rate, $r_{C_2H_3Cl}$ (mol g_{Ru}^{-1} s^{-1}), is calculated with $X_{C_2H_3Cl}$ as shown in the following equation.

$$r_{C_2H_3Cl}=\frac{N_{C_2H_3Cl}\times X_{C_2H_3Cl}}{g_{Ru}}$$

where $N_{C_2H_3Cl}$ is the VC gas flow rate (mol/s) and g_{Ru} is the weight of Ru (g) in the catalyst.

When the VC conversion is below 15%, a dependence of the reaction rate ($r_{C_2H_3Cl}$) on the products of CO_2 and H_2O may be ignored; hence, the empirical kinetic expression of the reaction rate equation of VC oxidation can be described as follows.

$$r_{C_2H_3Cl} = A \exp\left(-\frac{E_a}{RT}\right) P_{C_2H_3Cl}^{\alpha} P_{O_2}^{\beta}$$

Taking the logarithm of the above equation yields the following equation.

$$\ln r = \ln A + \alpha \ln P_{C_2H_3Cl} + \beta \ln P_{O_2} - E_a / (RT)$$

The components of the reactant feed gas are hardly changed during the kinetics testing with a low VC conversion below 15%. Therefore, $\ln A$, $\alpha\ln P_{C_2H_3Cl}$, and $\beta\ln P_{O_2}$ can be supposed to be approximately constant, and the equation can be simplified to $\ln r = -E_a / (RT) + C$. The activation energy (E_a) can be obtained from the slope of the resulting linear plot of ln r versus $1/T$.

The turnover frequency (TOF) was calculated to evaluate the intrinsic catalytic activity per exposed Ru atom, eliminating the interference of different amounts of accessible active sites among three catalysts. The relevant formulas are listed as follows:

The total molar amount of Ru loaded on the catalyst as shown in the following equation.

$$n_{\text{Ru,total}} = \frac{m_{cat} \times \omega}{M_{\text{Ru}}}$$

where m_{cat} is the mass of catalyst used for activity test, ω represents the Ru mass loading, and M_{Ru} is the molar mass of Ru.

The fraction of surface-exposed Ru atoms (f) was calculated via the formula below..

$$f = \frac{8r_{atom}}{d}$$

Here, r_{atom} is the metallic atomic radius of Ru, and d denotes the average diameter of Ru nanoparticles statistically counted from TEM images.

The molar quantity of surface active Ru sites can be described as follows.

$$n_{\mathrm{surf}} = n_{\mathrm{Ru,total}} \times f$$

Definition of turnover frequency (TOF) yields the following equation.

$$\mathrm{TOF} = \frac{r \times m_{cat}}{n_{\mathrm{surf}}}$$

3. Supplementary figures and tables

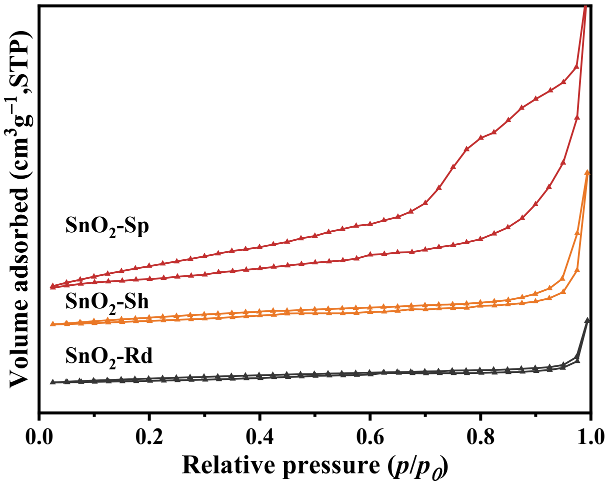

Figure S1. N_2 adsorption-desorption isotherms of SnO_2 supports.

Table S1. Catalytic activity comparison of the catalyst with those reported in the literatures.

Catalysts	VC concentration	T_{50} (°C)	T_{90} (°C)	WHSV (mL g^{-1} h^{-1})	*Reference*
Ru/SnO_2-Sp	0.1vol.%	244	268	15,000	This work
Ru/SnO_2-Sp	0.1vol.%	266	305	35,000	This work
Ru/SiO_2	0.1vol.%	258	277	15,000	[1]
Ru/Al_2O_3	0.1vol.%	271	291	15,000	[1]
1%Ru/CoPO-MCF	0.1vol.%	278	313	48,000	[2]
Ru/Sn-MFI	0.1vol.%	275	306	30,000	[3]
RuO_x/HZ5-3d	0.1vol.%	275	297	15,000	[4]
ZIF-CoCe	0.1 vol.%	244	275	15,000	[5]
Pt/TiO_2	0.1vol.%	280	325	15,000	[6]
Pt/ZrO_2	0.1 vol.%	283	313	15,000	[6]

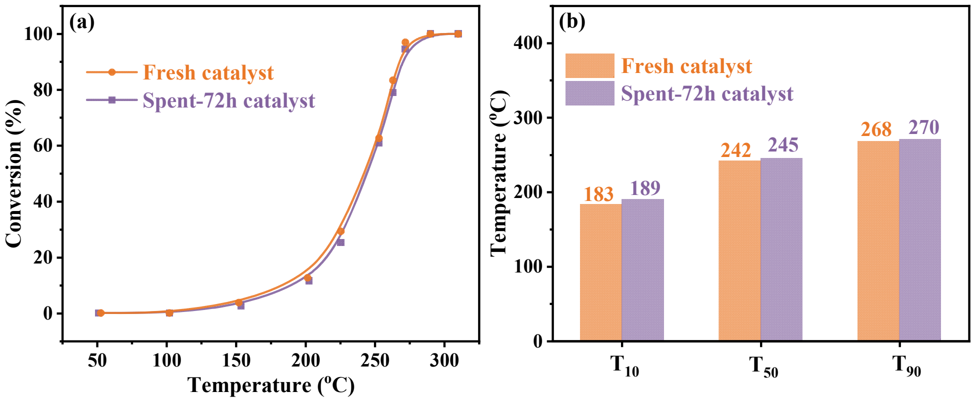

Figure S2. (a) Light-off curves as a function of reaction temperature and (b) the characteristic values of T_{10}, T_{50}, T_{90} over fresh Ru/SnO_2-Sp catalyst and spent catalyst after 72 h long-term stability test.

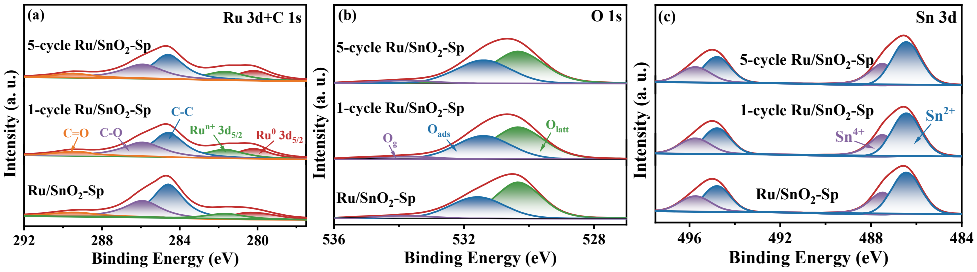

Figure S3. (a) Ru 3d + C 1s, (b) O 1s and (c) Sn 3d XPS spectra of the fresh, 1-cycle and 5-cycle Ru/SnO_2 catalysts.

Table S2. The detailed binding energies of O 1s and the quantitative results from XPS spectra deconvolution over the fresh, 1-cycle and 5-cycle Ru/SnO_2-Sp catalysts.

Catalysts	O 1s			Ru^0/Ru^{n+}	O_{ads}/O_{latt}	T_{90} (°C)
	O_{ads}	O_{latt}	O_g			
Ru/SnO_2-Sp	531.6	530.3	533.8	1.40	0.65	282
1-cycle Ru/SnO_2-Sp	531.4	530.3	533.8	0.98	0.72	265
5-cycle Ru/SnO_2-Sp	531.4	530.3	533.8	0.95	0.71	268

Table S3. Frequencies of functional groups present on the SnO_2-Sp and Ru/SnO_2-Sp catalysts analyzed by DRIFTs.

Wavenumber (cm^{-1})	Group	Reference
3780	δ(H–O–H)	[7]
2360	ν (CO_2)	[8]
1900	ν (O–H)	[1]
1600	ν (C=C)	[1]
1518	ν_{as} (COO)	[8]
1500	ν_{as} (COO)	[8]
1428	ν_{as} (COO)	[8]
1280	ν_{as} (C–H)	[5]
1250	ν_{as} (C–H)	[5]
1095	ν_{as} (COO)	[5]
800	ν_s (C–Cl)	[5]
654	ν_s (C–Cl)	[5]

Reference

1. Ding, M.; Zhang, Y.; Guo, Y.; Hua, W.; Yang, J.; Wang, L.; Guo, Y.; Dai, Q.; Wang, A.; Zhan, W. Selective adsorption of chlorine species on RuO_2 sites for efficient elimination of vinyl chloride on the Ru/SnO_2 catalyst. *Environ. Sci. Technol.* **2025**, *59*, 956-967, doi:10.1021/acs.Est.4c09658.
2. Wang, C.; Tian, C.; Guo, Y.; Zhang, Z.; Hua, W.; Zhan, W.; Guo, Y.; Wang, L.; Lu, G.; Ruthenium oxides supported on hetero-structured CoPO-MCF materials for catalytic oxidation of vinyl chloride emissions, J. Hazard. Mater. **2018**, *342*, 290-296, doi:10.1016/j.jhazmat.2017.08.036
3. Li, M.; Cai, Y.; Zhan, W.; Wang, L.; Dai, Q.; Guo, Y.; Wang, A.; Guo, Y.; Revealing the effect of framework acidity on the catalytic combustion of vinyl chloride over zeolite-based catalysts, Sep. Purif. Technol. **2024**, *340*, 126773, doi:10.1016/j.seppur.2024.126773
4. Wang, S.; Zhang, H.; Wang, M.; Liu, X.; Shang, S.; Wang, Z.; Zhang, C.; Micro-meso hierarchical ZSM-5 zeolite supported RuO_x nanoparticles for activity enhancement of catalytic vinyl chloride oxidation, Appl. Surf. Sci. **2022**, *606*, 154906, doi:10.1016/j.apsusc.2022.154906
5. Zhang, H.; Wang, S.; Wang, M.; Li, G.; Yu, L.; Liu, X.; Wang, Z.; Zhang, C. Catalytic oxidation of vinyl chloride over Co-Ce composite oxides derived from ZIF-67 template: Effect of cerium incorporation. J. Rare Earths 2023, 41, 870-880, doi:10.1016/j.jre.2022.12.006.
6. Wang, L.; Wu, Q.; Fang, T.; Ding, M.; Zhang, Q.; Wang, Z.; Guo, Y.; Zhan, W.; Guo, Y.; Wang, A. Boosting vinyl chloride catalytic combustion and chlorine resistance over Ti-modified Pt/ZrO2 catalysts. Sep. Purif. Technol. 2025, 372, 133536, doi:10.1016/j.seppur.2025.133536.
7. Liu, H.; Li, X.; Dai, Q.; Zhao, H.; Chai ,G.; Guo, Y. ; Guo, Y.; Wang, L.; Zhan, W.; Catalytic oxidation of chlorinated volatile organic compounds over Mn-Ti composite oxides catalysts: Elucidating the influence of surface acidity, Appl. Catal. B-Environ. **2021**, *282*, 119577, doi:10.1016/j.apcatb.2020.119577
8. Wang, M.; Li, G.; Wang, S.; Liu, X.; Wang, A.; Cao, H.; Zhang, C. Catalytic oxidation of propane over nanorod-like TiO_2 supported Ru catalysts: Structure-activity dependence and mechanistic insights. *Chem. Eng. J.* **2024**, *481*, 148344, doi:10.1016/j.cej.2023.148344.
